# Effects of verbal tasks on driving simulator performance

**DOI:** 10.1186/s41235-022-00357-x

**Published:** 2022-02-04

**Authors:** Jonathan C. Rann, Amit Almor

**Affiliations:** 1 Department of Psychology, University of South Carolina, 1512 Pendelton Street, Columbia, SC 29208 USA; 2 Institute for Mind and Brain, University of South Carolina, Columbia, SC 29208 USA; 3 Linguistics Program, University of South Carolina, Columbia, SC 29208 USA

**Keywords:** Cognitive load, Language processing, Multitasking, Simulated driving

## Abstract

We report results from a driving simulator paradigm we developed to test the fine temporal effects of verbal tasks on simultaneous tracking performance. A total of 74 undergraduate students participated in two experiments in which they controlled a cursor using the steering wheel to track a moving target and where the dependent measure was overall deviation from target. Experiment 1 tested tracking performance during slow and fast target speeds under conditions involving either no verbal input or output, passive listening to spoken prompts via headphones, or responding to spoken prompts. Experiment 2 was similar except that participants read written prompts overlain on the simulator screen instead of listening to spoken prompts. Performance in both experiments was worse during fast speeds and worst overall during responding conditions. Most significantly, fine scale time-course analysis revealed deteriorating tracking performance as participants prepared and began speaking and steadily improving performance while speaking. Additionally, post-block survey data revealed that conversation recall was best in responding conditions, and perceived difficulty increased with task complexity. Our study is the first to track temporal changes in interference at high resolution during the first hundreds of milliseconds of verbal production and comprehension. Our results are consistent with load-based theories of multitasking performance and show that language production, and, to a lesser extent, language comprehension tap resources also used for tracking. More generally, our paradigm provides a useful tool for measuring dynamical changes in tracking performance during verbal tasks due to the rapidly changing resource requirements of language production and comprehension.

## Statement of Significance

People often engage in verbal activities while driving. These can involve conversations with passengers in the car, cell phone conversations with people not in the car, or simply listening to the radio. Engaging in these multitasking activities has been shown to be detrimental to driving performance, and as a result, several studies aimed to elucidate what aspects of linguistic processing most heavily interfere with driving performance and to identify the cognitive and attentional mechanisms underlying this interference. In this article, we explore these questions with a novel driving simulator-based paradigm that allowed us to efficiently study the effect of language processing on performance on driving-based tracking tasks with sensitivity to the fine temporal changes in the demands of concurrent linguistic processing and with high level of experimental control. We performed two experiments which examined these effects when participants listened and responded to simple verbal tasks (E1), and when participants read and responded to presented text (E2). Our results were in line with current theories of speech production and language comprehension, as well as load-based theories of attention and multitasking performance. Overall, they show that language production, and, to a lesser extent, language comprehension tap similar resources as those used for tracking. More generally, our paradigm provides a useful tool for measuring the dynamical changes in driving performance during verbal tasks due to the rapidly changing resource requirements of language production and comprehension.

## Introduction

Drivers face many overlapping and often competing demands on their limited information processing resources while navigating the driving environment (da Silva, 2014; Metz et al., 2011; Regan et al., 2011; Young et al., 2007). This is especially the case when drivers concurrently engage in conversation (Bergen et al., 2013; Linardoua et al., 2018; Strayer & Cooper, 2015). In this scenario, drivers simultaneously operate and control the movement of a vehicle on a roadway (Fuller, 2005), and exchange verbal information with an interlocutor (Levinson & Torreira, 2015). As demands of the driving and verbal tasks increase, the ability of drivers to divide attention between tasks may degrade (Becic et al., 2010; Strayer & Drews, 2007; Strayer et al., 2015; Strayer, Biondi, et al., 2017; Strayer, Cooper, et al., 2017); this can result in an increased risk for fatal car crashes (National Center for Statistics and Analysis, 2021).

While there is a growing body of research aimed at testing and measuring the effects of conversation on driving performance (*for review*: Caird et al., 2018), the fine-grain dynamical performance trade-offs between driving and verbal communication (both auditory and text-based) remain unclear. This paper aims to elucidate these trade-offs with two driving simulator experiments that measured performance on a simple driving-based tracking task while drivers processed verbal input and generated verbal responses. Specifically, we examined how tracking performance changes dynamically during the course of conversational turns as drivers listen and verbally respond to prerecorded speech presented via headphones (Experiment 1), and read and verbally respond to text overlain on the driving simulator screen (Experiment 2). Being the first study to look at the interference between dialog-based verbal tasks and driving-based tracking performance at a fine temporal resolution, we are also able to relate the well-documented interference between conversation and driving to current literature in psycholinguistics and provide a detailed and psycholinguistically motivated model of the cognitive bases of this interference.

A primary goal of driving is to safely transport drivers, passengers, cargo, etc., from one location to another (Allen et al., 1971). To achieve this goal, drivers must perform a series of actions that allow them to control the lateral and longitudinal movement of the vehicle as they move through the driving environment. Michon (1985) characterizes these actions as a hierarchically structured set of interconnected problem-solving tasks. At the top of the hierarchy are actions involved with trip planning, goal setting, and analysis of risks and costs associated with the driving tasks (Dogan et al., 2011). Below that are highly skilled actions involved with non-routine maneuvers, such as the quick steering and braking responses required to avoid obstacles in the driving environment (Kaplan & Prato, 2012). Finally, at the bottom of the hierarchy are highly automatized actions involved with continuous driving behavior, such as the slow steering and braking responses required to maintain lateral lane position (Cooper et al., 2013) and headway (Brackstone & McDonald, 2007).

The driver-in-control (DiC) model (Hollnagel et al., 2003) expands on Michon’s (1985) model, organizing the driving task into hierarchical ‘loops’ in which control is shared in time (i.e., throughout the duration of the driving task). The higher-level loops, targeting and monitoring, both include actions that require anticipatory control, such as goal setting and assessment activities. The targeting loop is focused on the assessment of the driving situation over the course of the entire driving task (e.g., determining best path to destination), whereas the monitoring loop focuses on immediate driving goals (e.g., swerving to avoid collision). In contrast, the lower-level loops, tracking and regulating, both include actions which require more compensatory control. The tracking loop mainly involves driving actions (e.g., continuous steering), whereas the regulating loop provides the criteria and goals for those actions (e.g., staying within designated lane).

According to the DiC model (Hollnagel et al., 2003), driving performance reflects drivers’ ability to simultaneously maintain control over the multiple loops at any given time. For example, drivers must establish the proper positioning and velocity criteria (i.e., regulating) in order to maintain lane position using the steering wheel (i.e., tracking). Similarly, drivers must attend to traffic signs, signals, and other stimuli that they encounter along the way (i.e., monitoring) in order to strategize and adjust their plan during their journey through the driving environment (i.e., targeting). Because the focus of our research is on how regular routine driving is affected by simultaneous conversation, we focus on the lower-level loops that are constantly engaged during continuous routine driving.

Underlying these control loops are information processing mechanisms which, during driving, support drivers’ ability to focus on and process task-relevant perceptual stimuli within the driving environment, while ignoring task-irrelevant stimuli (e.g., Engström, 2011; Strayer & Fisher, 2016). How and when perceptual stimuli are selected for higher-level processing is a matter of debate in the broader cognitive psychology literature about attentional selection. Early work by Broadbent (1958) argued that since perceptual capacity is limited, selection occurs early during perception based on only some salient physical aspects of stimuli. Other theories have instead argued for the late selection of relevant stimuli on the basis of not only the stimuli’s physical properties but also its meaning (e.g., Deutsch & Deutsch, 1963; MacKay, 1973; Treisman, 1964). For example, cognitive relevance theory (Henderson, 2017; Henderson et al., 2009) explains that meaning plays a larger role than salience in guiding attention selection during the processing of real-world visual scenes, such as those encountered while driving.

Remarkably, there is considerable empirical evidence in support of both early and late selection. To explain these seemingly contradictory results, Lavie et al. (2004) proposed the load theory, which argues that both ‘low-level’ perceptual selection and ‘high-level’ cognitive control mechanisms play integral roles in selective attention and the ability to reject distracting stimuli. According to the theory, perceptual selection mechanisms allow for the reduction of distractor interference effects during high perceptual load scenarios, resulting in behavior that is consistent with early selection. These are considered to be passive mechanisms in that irrelevant stimuli are simply ignored when limited perceptual capacity is exceeded and is therefore not available for processing distractors. In contrast, cognitive control mechanisms actively reject perceived stimuli based on processing priorities managed and maintained by central executive and other higher cognitive functions. High load on these cognitive control processes should deplete active control resources, thus resulting in reduced selection which will in turn lead to increased processing of distracting stimuli, consistent with late selection.

With regard to driving, both the selection of relevant stimuli and the processing of distractor stimuli can be greatly affected by the demands of the tasks that drivers perform (Engström et al., 2017; Lee et al., 2009). For example, the tracking and regulating required to maintain lateral lane position may normally be minimally demanding when performed in the absence of secondary distraction (Laberge et al., 2004). However, maintaining lane position may become more difficult when the demands of the driving task increase, for example, when the speed of the driving task increases (Aarts & Van Schagen, 2006), and when drivers concurrently engage in a demanding secondary task, such as conversation. In-line with Lavie et al. (2004), we reason that increased demands may have different effects on certain measures of driving performance depending upon whether these demands overload perceptual selection or cognitive control mechanisms (Murphy & Greene, 2017). In the former case, processing a secondary task, such as conversation, may have less of an effect on driving performance since drivers might have fewer resources available to process distraction while driving. In the latter case, processing a secondary task may have more of an effect on performance since drivers might not have enough resources available to actively reject distracting stimuli such as conversation. As our focus here is on understanding the reasons for the well-documented interference between conversation and driving, it is necessary to explore the processes underlying the different aspects of verbal exchange that may make conversation either perceptually or cognitively demanding.

Conversation is a demanding activity in which interlocutors exchange and process verbal information (Clark, 1996). During these exchanges, linguistic signals can take many forms, such as spoken and heard utterances during spoken dialogue (Barthel et al., 2016). In spoken conversations, listeners first identify, decode, and derive meaning from auditory verbal signals (MacDonald & Hsiao, 2018). Then, as they prepare for their speaking turn, they must plan and decide on what information they want to express, and compose and encode it into a properly formed message (Ferreira, 2010; Ferreira & Swets, 2002; Levelt, 1999; Roelofs et al., 2007). Finally, when their turn approaches, they must monitor the planned output (Levelt, 1989; Nozari & Novick, 2017), and then, if no corrections are required, vocally articulate it into a linear sequence of utterances (Ferreira & Henderson, 1998; Lee et al., 2013; Levelt, 1981, 1982; Postma, 2000).

The demands of each language process can vary depending on the mechanisms engaged during their execution (Lee et al., 2017). For example, speech comprehension is thought to involve parallel processes which normally create quick, superficial interpretations which are continuously weighed and revised on the basis of probabilistic constraints (Ferreira & Lowder, 2016; Ferreira et al., 2009; Ferreira & Henderson, 1991; Ferreira & Patson, 2007; MacDonald, 2013; Seidenberg & MacDonald, 2001). Speech planning is thought to involve controlled processes that are more sequential (although not necessarily strictly sequential) for message planning and composition (Barthel & Sauppe, 2019; Dell, 1986; MacDonald, 2016; Roelofs & Piai, 2011; Swets et al., 2014) and is subject to time constraints imposed by the need to provide unique interpretable output during quick conversation turns (Sjerps & Meyer, 2015). Finally, speech production is thought to involve highly controlled processes for monitoring and error-checking (Ferreira, 2019), audience design (Horton & Gerrig, 2005), and speech articulation (Alario et al., 2006). Therefore, although speech comprehension may require considerable resources (e.g., Caplan & Waters, 1999; Just & Carpenter, 1992), these requirements are not likely as high as in speech planning and production which require quick commitments to a single specific output that is to be produced (Kubose et al., 2006).

The demands of language processing can further increase due to the need for managing conversational turns (Pickering & Garrod, 2013). While conversational turns may appear sequential and non-overlapping (e.g., listeners listen as speakers speak; Hoey & Kendrick, 2017), interlocutors often speak at the same time, interrupt each other, and pause for variable lengths during vocal conversation (Fusaroli & Tylén, 2016; Gravano & Hirschberg, 2012; Heldner & Edlund, 2010; Yuan et al., 2007). Moreover, interlocutors often overlap specific language processes, such as when both listeners and speakers simultaneously plan their next contributions and anticipate upcoming conversation turns (Garrod & Pickering, 2009; Levinson, 2016). Therefore, these characteristics, which are quite typical of conversation, can increase processing demands during verbal exchanges (Bock et al., 2007). Importantly, all the psycholinguistic processes described so far occur at a very fine time scale, at the order of magnitude of up to a few hundreds of milliseconds and often much less than that (Bock, 1996; Garrod & Pickering, 2009; MacDonald & Hsiao, 2018).

The modality of the verbal exchange can also affect the demands of language processing (Schaeffner et al., 2016). Like speaking and listening, writing and reading also involve language production and comprehension (Parodi, 2007). Whereas the production of speech requires processes which transform intended messages into vocal articulations (as discussed above), writing text requires processes which transform intended messages into manual motor gestures (Hayes, 2012). Similarly, as the comprehension of speech involves the parsing and decoding of auditory stimuli into comprehended meaning, reading text involves the parsing and decoding of visual script into meaning (Rapp & Van Den Broek, 2005). Although many commonalities exist between both sets of production and comprehension processes (Cleland & Pickering, 2006; Gullberg, 2020; Hayes & Chenoweth, 2006; Jobard et al., 2007; Rayner & Clifton Jr., 2009), the involvement of mental speech simulations (i.e., inner speech) (Emerson & Miyake, 2003; Perrone-Bertolotti et al., 2014), as well as less restrictive time constraints (Auer, 2009; Boland, 2004), may result in differing levels of demand on attentional resources while using language in the two modalities (Conners, 2009; Olive et al., 2008).

Regarding driving, our concern is primarily with listening to speech, planning and producing speech, and reading text, since writing text while driving is clearly disruptive because, in addition to occupying cognitive resources, it requires one or both hands and loads the visual system while also drawing attention away from the road environment to a handheld device (a trivial fact which, while seeming to be lost on the many drivers who text while driving, hardly needs any scientific support) (Caird et al., 2014a, 2014b; He et al., 2015). When drivers concurrently engage in conversation, they must carefully balance the demands of listening, planning, speaking, and reading as each of these may interfere with driving performance (Salvucci & Beltowska, 2008). However, while the processes underlying the comprehension of language (both speech and text) are thought to be less demanding on attentional resources than those involved with speech planning and production (Bergen et al., 2013; Christodoulides, 2016; Kubose et al., 2006), these differences are not well addressed in the dual-tasking literature involving driving and conversation. In particular, since people switch rapidly between comprehension, speech planning and production, any examination of the mechanisms underlying the interference between verbal tasks and driving should focus on dynamic changes that occur on a time scale of less than a hundred milliseconds (Laganaro et al., 2012). A useful cognitive framework to capture the interplay between the demands of driving and verbal tasks as described so far is provided by Wickens’ (2002) model for resource competition during dual-task scenarios, which we describe next.

Wickens (2002) proposed a model in which four dichotomous dimensions are used to predict consequences of concurrent task performance by determining the demand for separate and shared resources between particular tasks. These dimensions include: processing stages (perception/cognition and response selection/execution), perceptual modalities (visual and auditory senses), vision channels (focal and ambient vision), and processing codes (spatial and symbolic processes). Accordingly, this model predicts that as the number of dimensions shared between concurrent tasks increases, performance on the tasks degrades. For example, concurrent visuo-spatial and audio-verbal tasks would operate in different dimensions, resulting in less interference than concurrent visuo-spatial and audio-spatial tasks, which overlap in one dimension.

Applying Wickens’ (2002) model to the specific situation of driving while performing a verbal task reveals attentional resource allocation shared between modalities, spatial codes, and processing stages. For driving, drivers use their vision (and to a much lesser extent their hearing) to continually perceive the driving environment, while taking into account spatial relations for safe maneuvering, successful vehicle navigation, and responding when necessary to environmental stimuli (Horrey et al., 2006). When the difficulty of the driving task increases, higher demands are placed on these resources. For verbal tasks, listening to speech places varying amounts of load on the auditory perceptual modality, while producing speech places load on motor resources associated with articulating and monitoring language. Planning speech places load on cognitive processes and motor resources associated with planning vocal responses (Ferreira & Swets, 2002; Silveri & Misciagna, 2000), especially when this planning involves the memorization of topics discussed by the conversation partner that will soon need to be addressed in a later conversation turn (Almor, 2008). This is further complicated by the fact that different aspects of language processing do not operate in strict sequential fashion but instead overlap (Dell et al., 1997; Levelt et al., 1999), thus resulting in magnified demands of cognitive resources.

Reading written or typed text places load on the visual perceptual modality. According to Wickens (2002), when drivers concurrently engage in reading activities (e.g., reading text messages from cellphone, reading billboards, etc.), attentional load is further increased due to the overlap between the visual resources needed for the incremental recognition and comprehension of text, and the visual attentional resources required for driving. Thus, reading text should cause more noticeable interference on the driving task compared to listening to speech.

While the Wickens’ (2002) multiple resource model provides a useful means of characterizing the sources of interference produced when drivers concurrently engage in conversation, it does not account for the dynamically shifting demands of conversational exchanges over the course of a driving task. After all, driving and conversation are both activities that take place in time (Watson & Strayer, 2010), and thus involve the performance of tasks that vary in sequence, duration, and frequency of execution (Hollnagel et al., 2003; Salvucci et al., 2009). To address this, Salvucci and Taatgen (2008) presented threaded cognition, an integrated theory of multitasking implemented within the ACT-R cognitive framework (Anderson et al., 2004).

According to the theory, task goals (e.g., driving, listening, etc.) can be represented as independent ‘threads’ consisting of interleaving blocks of rule firings in which distinct cognitive resources (e.g., perceptual, cognitive, motor, etc.) are requested as needed and used as made available by a central procedural resource every 50 ms. During concurrent multitasking, several threads can be active at once, but a particular resource can only be used by a single thread at any given time. Unlike other theories of multitasking (e.g., Kieras et al., 2000; Meyer & Kieras, 1997), threaded cognition does not require an executive which assigns available resources to threads (Borst & Taatgen, 2007). Instead, resources are shared in a greedy/polite manner in which a thread can claim any available resource (greedy) but will immediately release it once they are done with it (polite). Further, least recently processed threads are favored by the procedural resource to balance task execution. Regarding performance, interference during multitasking can arise from peripheral bottlenecks involving visual and motor resources (Wickens, 2008), and central bottlenecks involving declarative and procedural memory (Borst et al., 2010; Marti et al., 2012; Pashler, 1994). However, this interference can be reduced with practice (Koch et al., 2018).

To test the predictions set forth by threaded cognition, Salvucci and Taatgen (2008) utilized the ACT-R Integrated Driver Model (Salvucci, 2005, 2006), which itself is based off the core components described in Michon’s (1985) model of driving. The model describes the continuous steering behavior involved with several driving tasks (e.g., lane maintenance, curve negotiation, etc.) as a running calculation in which drivers continuously update the steering wheel angle using two visual points: a near point which helps with maintaining lane position within lane boundaries, and a far point which helps drivers anticipate changes in the roadway (Salvucci & Gray, 2004). Within threaded cognition, this model of driving was implemented as a set of rules that continuously iterated in sequence, and updated steering angle and acceleration after each iteration.

The authors integrated the driving model into several multitasking studies involving verbal tasks from different modalities. For example, the ‘driving and sentence-span task’ was based on the study presented in Alm and Nilsson (1994) in which drivers followed a lead vehicle and concurrently engaged in a cognitively intensive secondary language task in which they judged the sensibility of a presented sentence and memorized the final words through reading and speaking (Daneman & Carpenter, 1980; Lovett et al., 2000). Further, the ‘driving and dialing task’ was based on the driving simulator study presented in Salvucci (2001) in which drivers steered to maintain lane position as their vehicle moved at a constant speed and dialed a phone number via manual entry and voice command. Overall, the results of these studies showed that the integrated driver model was successful in capturing curve negotiating and lane positioning behavior exhibited by drivers under controlled experimental conditions (Salvucci et al., 2001). However, no study has looked at the fine-grain temporal dynamics of the interference between driving and a verbal task to see whether it reflects the production and comprehension processes identified by psycholinguists.

In summary, drivers use their limited attentional resources to continuously manage the visuo-spatial and motor processing demands required by the driving task (Strayer, Biondi, et al., 2017; Strayer, Cooper, et al., 2017; Wickens, 2002). Often, drivers engage in conversational activities in which they take turns producing and comprehending language with an interlocutor (e.g., passenger in the car, friend calling from cell phone). They also engage in unidirectional language-based activities, such as when they listen to the radio without producing verbal responses (e.g., Strayer & Johnston, 2001). These secondary language tasks have their own resource requirements depending upon the specific operations performed in the task. For example, listening to speech taps auditory-cognitive resources used for decoding and interpreting verbal input (Diehl et al., 2004), while reading text taps visual-cognitive resources used for decoding textual input (Rapp & Van Den Broek, 2005). Further, producing speech taps a-modal central executive resources for message planning, motor planning resources for utterance planning, and then actual motor resources for utterance articulation (Levelt, 1999).

Several studies have shown that planning and producing speech causes more interference on the driving-like tasks than comprehending speech. This was shown to be the case for both ball tracking (e.g., Almor, 2008) and driving simulator-based tasks (e.g., Strayer et al., 2003), and for both artificial (e.g., Beede & Kass, 2006) and naturalistic (e.g., Boiteau et al., 2014) verbal tasks. What remains unclear is: (1) whether the interference between verbal tasks of different modalities and driving performance under different difficulty conditions is compatible with the theoretical analysis provided here, and (2) whether this interference follows the fine-grain temporal dynamics predicted by psycholinguistic models of language comprehension, production, and dialogue.

We explore these questions using a novel driving simulator paradigm which allows for the testing of the effects of verbal tasks on driving-based tracking performance with a high level of experimental control and with sensitivity to the fine temporal changes in the demands of concurrent linguistic processing. This paradigm is based on the OpenDS driving simulator platform (Math et al., 2012), and the continuous tracking and reaction (ConTRe) task (Mahr et al., 2012) implemented in the simulator. The ConTRe is a pursuit tracking task in which participants use a steering wheel peripheral to align a cylindrical indicator with a smoothly moving target within the driving environment. The dependent measure is the average distance between the driver-controlled cursor and the moving target. We chose this task because it provides a good proxy of a critical aspect of basic routine driving, namely continuously controlling the lateral position of the vehicle while driving, because it provides temporally fine-grain data about driving performance, and because it was previously used to investigate the interference between driving and language (Demberg, 2013; Häuser et al., 2019; Rajan et al., 2016; Vogels et al., 2020). This allowed us to measure the effects of a concurrent interactive verbal task at a high temporal resolution and thus provide a critical test of a psycholinguistic explanation of the well-documented interference between conversation and driving. While this task was used before to test the effects of linguistic complexity (e.g., Demberg & Sayeed, 2016) and structural ambiguity (e.g., Demberg et al., 2013) on concurrent driving, we use it here for the first time to study the unique requirements of production and comprehension in the context of an interactive verbal task.

The two experiments we report are similar to Boiteau et al. (2014) in providing high temporal resolution analysis of the interference between processing language and tracking performance but are different in employing a driving simulator and in examining both written and spoken verbal input.

## Experiment 1

Experiment 1 (E1) tested participant performance on a driving simulator-based tracking task during fast and slow target speeds (Fast and Slow conditions) and under conditions involving no verbal input or output, conditions with passive listening to spoken prompts via headphones and conditions in which participants responded to the prompts they heard (Absent, Listen and Respond conditions). At the beginning of the experiment, participants were informed that, at the end of each experimental block that included verbal input, they will be given a memory task about the verbal stimuli in the block. This task served to both ensure that participants actively engaged with the verbal stimuli during each block, and to assess their retention of the verbal information. We also asked participants for their perceived level of difficulty after each block of the experiment. We start by describing our most important hypotheses and then review the less surprising predictions.

Our first critical hypothesis (H1) is that tracking performance should change dynamically throughout the course of conversational turns. This hypothesis follows directly from our analysis of language production being more demanding than language comprehension due to production’s greater requirements for quick responses and cognitive resources for planning and monitoring. Therefore, during listening segments, performance should be best at the beginning and then gradually worsen as participants memorize what they heard or plan their response. During talking segments, performance should be worst at the beginning and then improve as participants disengage planning in preparation for the other person to speak. These effects should be stronger in responding blocks when participants have to form verbal responses than in listening blocks when they only have to memorize what they heard.

Our second critical hypothesis (H2) is that variation in tracking and recall performance due to conversation complexity should reveal whether the load associated with increased tracking speed is perceptual or cognitive. This follows from attentional resource theories which state that performance on concurrent tasks such as driving and conversation may vary based on both the amount and type of load placed on perceptual and cognitive attentional resources (Lavie, et al., 2004; Salvucci & Taatgen, 2008; Wickens, 2002). From this perspective, if fast tracking speeds increase perceptual but not cognitive load relative to slow speeds, differences in performance due to conversation difficulty should be more noticeable when tracking speeds are slow compared to fast; this could be attributed to fewer attentional resources available for processing conversation during fast tracking thus resulting in reduced effects of conversation complexity on tracking performance. Alternatively, if fast speeds increase cognitive and not perceptual load relative to slow speeds, differences in performance due to conversation difficulty should be less noticeable in slow compared to fast speeds, which can be attributed to more cognitive resources being available for processing distracting conversation in slow speeds.

We also make several general predictions based off current theories of attentional resource allocation (e.g., Lavie et al., 2004; Wickens, 2002), as well as theories relating to resource demands of speech production (e.g., Ferreira & Pashler, 2002; Roelofs & Piai, 2011) and comprehension (e.g., Hauk et al., 2008). First, due to the increased demands placed on attentional resources during fast target tracking, we predict that performance would be worse overall in the fast target conditions than in the slow ones. Further, Almor (2008) and Boiteau et al. (2014) showed that visuo-motor task performance was worse when planning and producing compared to listening to speech. Therefore, we predict that the combination of verbal tasks and target tracking at different speeds should result in performance being best when no conversation is present, second best when listening to speech and worst when responding to speech. Using similar logic, we also predict that perceived difficulty would be worst overall in fast compared to slow speeds, and that, more interestingly, it would be lowest in the absence of any conversation, higher when only listening to verbal input, and highest when also having to respond verbally to the verbal input. Because our focus in this paper is on driving-based tracking performance, we avoid making predictions about the results of the memory recall task whose main function was to encourage participants to process the linguistic material.

### Methods

#### Participants

A total of 43 native English-speaking participants (age: *M* = 21, *SD* = 5.2) from the University of South Carolina Department of Psychology undergraduate participant pool took part in the study. Of the 43 participants, seven were male (age: *M* = 19.29, *SD* = 0.89) and 36 female (age: *M* = 21.13, *SD* = 5.67). Participants were compensated with extra credit for their time and signed an informed consent approved by the University of South Carolina’s IRB before the start of the experiment. Participant recruitment criteria specified that participants had to be native speakers of English and review of video recordings of the experiments confirmed that all spoke English with no foreign accent and at a level of native speaker. We did not collect data about participants’ driving experience. However, pilot experiments with the same population indicated that the vast majority of students in the participant pool have driving experience. There were no other inclusion or exclusion criteria for selecting participants.

#### Hardware

Microsoft SideWinder Precision Racing Wheel (USB) driver interface was used for steering wheel and foot controls. The driving simulator was run and presented on a Dell Desktop Computer running Windows 10 Pro with a 27″ full HD 1920 × 1080 flat panel monitor. Conversation tasks were presented via headphones. Experiment sessions were video recorded using LogiTech C920 HD Pro Webcam with a microphone. The purpose of these recordings was to ensure that participants complied with the experiment requirements and performed the task as expected.

#### Driving simulator

The OpenDS Driving Simulator (Math et al., 2012) was used to implement this experiment. OpenDS is an open-source simulation software specifically designed for the research and evaluation of driver behavior. The software provides an accurate physical environment with realistic forces, lighting, and road conditions that can be customized and configured for many types of scenarios. In our experiment, there were no road signs or any other roadside objects programmed into the script. Every detail of the driving simulation is described in xml files which are loaded into the software upon initialization. During the execution of a particular task, continuous measures of performance are recorded, thus providing measures of time, position, events, and other parameters at a high temporal resolution of approximately one measure per 19 ms. Once the tasks were completed, OpenDS stored task data into MySQL database for later analysis.

#### Procedure

After signing the consent form, participants were given instructions for the experiment and were then placed approximately 2 feet in front of a computer monitor with an attached steering wheel. This setup replicated an actual car driving experience for the seated participant. Next, a video recorder was turned on before the experiment began. The purpose of the video recordings was to ensure that participants fully complied with each task condition (e.g., consistently looked at the screen, verbally responding when required, and not responding when not required).

Before each experiment block, the researcher ran a batch file which set the variables and parameters for the driving simulator for the next block. Each block represented a unique combination of the target speed and conversation experimental conditions (Fast vs. Slow and Absent vs. Listen vs. Respond). Participants were first required to complete a practice session consisting of four blocks, with each block lasting approximately 30 s for a total of two minutes. The purpose of the practice session was to help acclimate participants to the driving-based tracking task in the simulator environment and to prepare them for the actual conditions presented in the experiment. The order of the practice blocks was as follows: Slow-Absent, Fast-Absent, Slow-Listen, and Slow-Respond. At the end of practice, participants completed a post-practice survey similar in form to the one they would have to fill out at the end of the experiment.

After completion of the practice blocks, the participants began the experiment, which was composed of six blocks, each lasting approximately four minutes. Each block included a unique combination of the levels of the target speed and conversation conditions. Five random block order lists were created, and each participant was randomly assigned to one of these lists.

#### Conversation task

During conversation blocks (i.e. Listen and Respond), participants heard 12 prerecorded statements at a rate of about one per every 20 s via headphones attached to the computer running the experiment. The precise onsets of the statements were jittered to prevent participants from predicting when each will be heard. The prerecorded statements were of people stating their name, occupation and place of employment, such as “Hello my name is Steve and I am an accountant at Bank of America.” During the Listen conditions, participants were tasked with actively listening to the prerecorded statements and trying to remember the information heard while performing the primary tracking task. During the Respond conditions, participants were required to actively listen to the prerecorded statements and then respond as if they were greeting the person in the statement by repeating what they heard as best as possible. For example, when the participant heard the prerecorded statement above, they were instructed to respond by saying “Hi Steve, accountant at Bank of America.” There were 48 recordings of both male and female voices The mean duration of these statements was 4395 ms (*SD* = 771.58). The recordings were presented in the same order for each participant.

#### Visuo-motor task

**Fig. 1:**
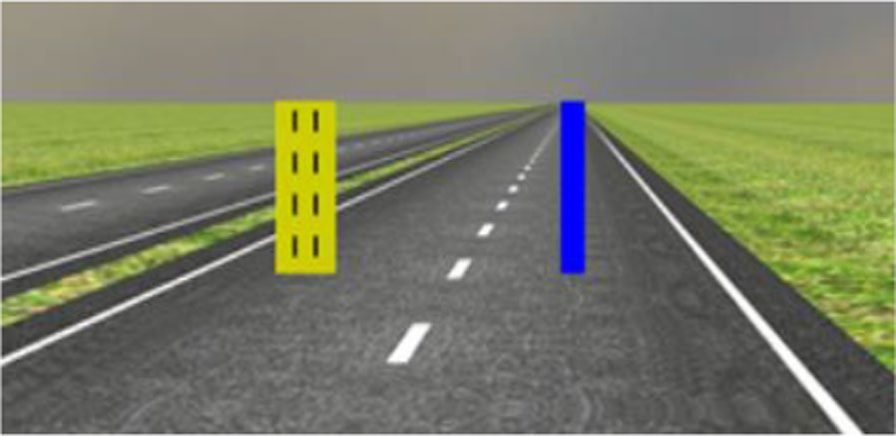
The indicator (blue cylinder) is controlled by the participants using the steering wheel as the target (yellow cylinder) moves laterally in the ConTRe task (Mahr et al., 2012). Both move forward at a constant speed to simulate a driving experience

The continuous tracking and reaction (ConTRe) task (Mahr et al., 2012), implemented as part of the OpenDS driving simulator, was the primary driving-based task used to measure tracking performance. In this task, participants are instructed to track the movement of yellow target cylinder, placed approximately 20 ft in front of the participants’ view, with a blue cylinder they control using the steering wheel. The yellow cylinder moves horizontally (i.e., left-to-right, right-to-left) across the screen at constant lateral speed of 1 m per second during Fast conditions and 0.4 m per second during slow conditions. The yellow cylinder’s direction of movement (left vs. right) changes at random times. Participants only have control of the lateral movement of the blue cylinder. Performance in this task is measured as the overall lateral distance in simulated meters between the driver-controlled cylinder and moving yellow cylinder during each experiment block (Fig. 1).

#### Post-block survey

Perceived block difficulty was recorded after each experiment block using a five-point Likert-like scale. A cued recall memory task was administered at the end of each Listen and Respond condition that listed the 12 statements presented to participants during the previous block. Each of the statements had either the name, occupation, or place of employment blanked out, and participants were required to recall and write down the missing information. Performance was scored as the total number of correct responses. Participants were told about these surveys at the beginning of the experiment and took the first survey at the end of the practice block. Recall performance was graded. Both perceived difficulty and survey data were analyzed after the experiment.

#### Data preparation

Upon the completion of each block, the data from that block were automatically stored into a MySQL database. Once all data (from all experiment blocks for all participants) were collected, it was exported from MySQL and converted to comma-delimited-value files via a SQL 5.7 script for statistical analysis. Next, the video recordings were examined to ensure participants’ compliance. Incompliance was defined as subjects speaking during Absent or Listen blocks, not speaking during Respond blocks, writing down answers while tracking and not attending the tracking task. To avoid any artifacts of starting or ending a block, five seconds of performance data from the beginning and end of each block were removed. The performance data were then segmented into Listen and Respond segments. Listening segments consisted of data recorded between the onsets and offsets of the audio prompts. Memorizing segments consisted of data recorded between the offsets of the audio prompts and approximately 4.5 s after their offset in memorize blocks. Speaking segments consisted of data recorded between the same boundaries in Respond blocks. In both blocks, data tagged as None segments consisted of the remaining data not associated with these three.

Reponses from the end-of-block recall surveys were scored as correct if they matched the missing information from the statement participants heard in the previous block. Responses that were similar to the correct response but did not repeat it verbatim were considered correct (e.g., listing Charlie instead of Charles for the missing name field). Responses matching information heard by the participant in a different trial than the target trial were counted as incorrect. Recall accuracy was calculated as the ratio of correct responses to the overall number of items in the block which was 12.

### Results

Data from 12 participants were removed due to lack of compliance. In addition, data from one participant were removed due to technical issues. Data from the remaining 30 participants (age: *M* = 21, *SD* = 6.2) were submitted for further analysis. Of these, five were male (age: *M* = 19, *SD* = 1) and 25 female (age: *M* = 22, *SD* = 6.7). This distribution is typical for the psychology undergraduate participant pool at the University of South Carolina. All analyses were performed in R 3.5.0 (R Core Team, 2018).

#### Overall analysis

**Fig. 2:**
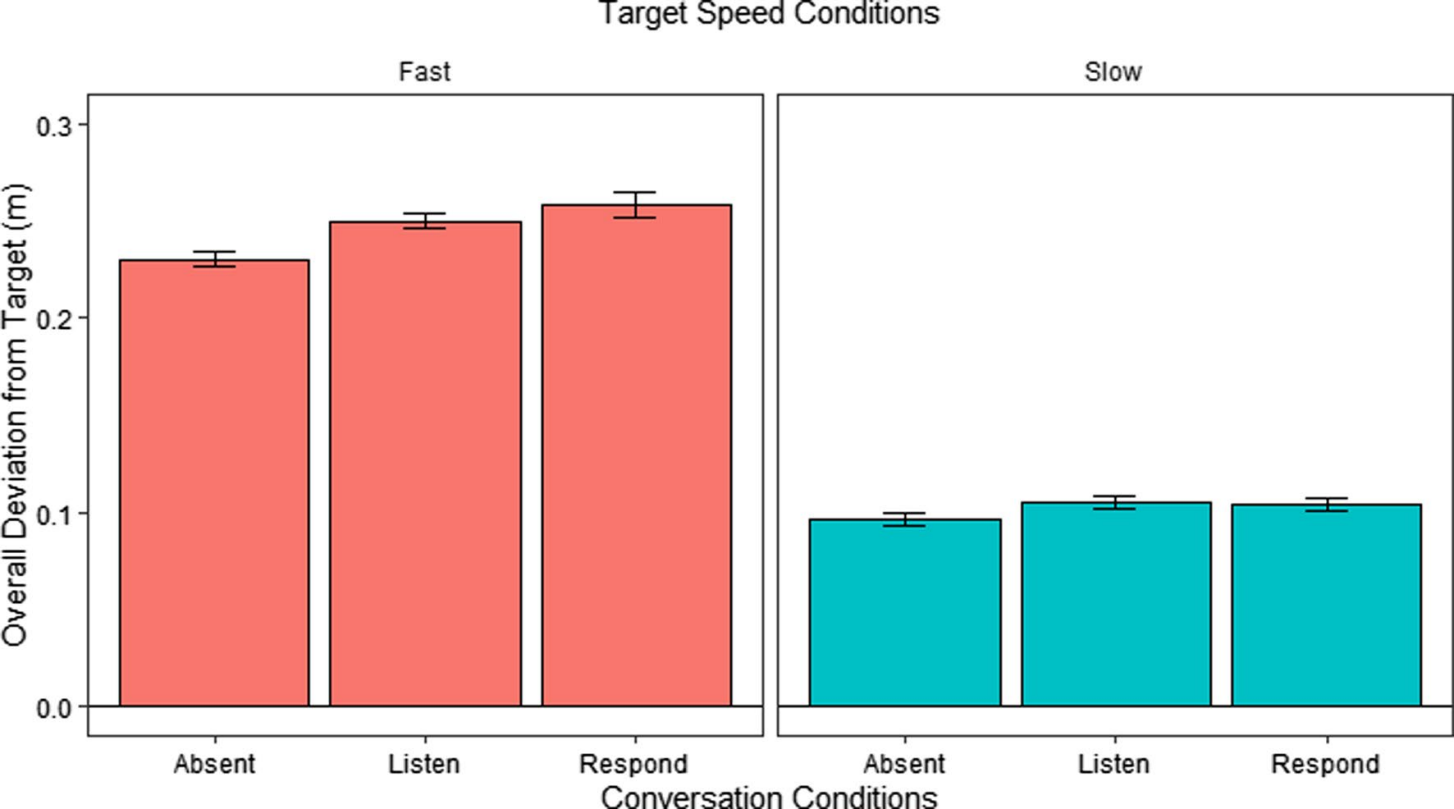
Overall deviation from target during different conversation conditions in Fast and Slow speed conditions in E1. *Error bars* show the standard error of the mean (SEM)

Figure 2 shows the overall absolute deviation in meters from target (deviation) in the different conversation conditions for Fast and Slow speed conditions. We analyzed these using a repeated measures ANOVA with speed and conversation set as within-subject factors and found significant main effects of both speed, *F*(1, 29) = 917.56, *p* < 0.001, and conversation, *F*(2, 58) = 12.96, *p* < 0.001, as well as an interaction between speed and conversation, *F*(2, 58) = 3.87, *p* = 0.03.

To better understand the nature of the 2 × 3 interaction, we followed up with Bonferroni corrected post hoc comparisons of performance in the conversation conditions separately for the Slow and Fast conditions. For the Fast conditions, there were significant differences between the Absent and Listen conditions, *t*(116) = − 3.77, *p* = 0.002, and Absent and Respond conditions, *t*(116) = 5.41, *p* < 0.001. The difference between Listen and Respond was not significant, *t* < 2. In the Slow conditions, there were no significant differences in any of the pairwise comparisons, *t*’s < 2.

This pattern of results shows that engaging in a verbal task affects tracking performance under difficult conditions (Fast conditions) more than under easy conditions (Slow conditions). This is reflected both in the overall difference in performance between the conversational conditions under the Fast conditions as well as by the post hoc differences between the Absent condition and both the Listen and Respond conditions only in the fast but not in the low speeds. In this analysis, however, there were no differences between the Listen and Respond conditions. This lack of difference may indicate that an analysis of the data from the entire block may not be sensitive enough as the blocks contain significant portions without verbal stimulation, during which the Listen and Respond blocks are essentially identical. Our next analysis focuses on only the times that involve listening or memorizing or speaking in response to verbal stimulation and may therefore be more apt to reveal subtle effects of conversation condition.

#### Time-course analysis

In order to test the effects of speed and conversation on performance across time, we utilized growth curve analyses (GCAs), following the procedure used in Boiteau et al. (2014). In preparation for the GCAs, we first extracted data from the conversation segments (i.e., Listening segments in Listen and Respond blocks; Memorizing segments in Listen blocks; and Speaking segments in Respond blocks). Data from the Absent blocks and from None segments in the other blocks were not included in this analysis. Due to the short duration of each event during conversation conditions (i.e., mean duration approximately 4.5 s), we chose to only look at performance over the first 2500 ms (i.e., 133 samples) of each segment onset. The reason for choosing this time interval was that prespeech planning takes about 1.5 s (Gleitman et al., 2007; Griffin & Bock, 2000), and since we wanted to include in our interval both the planning and the initiation of actual speaking, we extended this interval to 2.5 s. Then, using the R package lme4 version 1.1-17 (Bates et al., 2014), we fit the data using multilevel regression models that included Speed (Fast vs Slow), Block (Listen vs. Respond), Segment-type (Listening vs. Responding/Memorizing), and terms representing time.

To account for potential nonlinear changes in tracking performance across time, all models included baseline linear (i.e., Time^1^), quadratic (i.e., Time^2^), cubic (i.e., Time^3^), and quartic (i.e., Time^4^) time terms, as well as a random participant intercept term and a random participant slope term for speed. In this type of model, all time terms have the same number of bins (133 in our case). We also attempted to fit models with more complex random factor terms to the data, but these models did not converge. We first fit the data with a base model that only included the baseline time terms and the random factors but no fixed terms representing our conditions (Model 1 in Table 1, in Appendix). We then gradually added fixed terms representing the interaction of Conversation, Speed, and Segment-type with different time order terms (Models 2 – 6 in Table 1, in Appendix). We then used maximum likelihood estimates and Akaike information criterion (AIC) (Long, 2012) for model comparison to determine the best time order model to use. More complex models were preferred over simpler ones if the p value for the maximum likelihood test was smaller than 0.1. Table 2 shows the selection criteria for the models. Following Long (2012), we then interpreted the chosen model by looking at the coefficients together with visually inspecting the plot of the fitted model.

**Table 1:** Growth curve models for fitting distance from target for subject *i* at time point *j*

Model	Equation
1. Base	*Ƴ*_*ij*_ = *β*_0*i*_ + *β*_1_ * Time_*j*_ + *β*_2_ * Time_*j*_^2^ + *β*_3_ * Time_*j*_^3^ + *β*_4_ * Time_*j*_^4^ + *ε*_*i*_
2. Intercept	*β*_0*i*_ = *ζ*_0_ + *ζ*_0*i*_ * Speed *β*_0_ = *ζ*_1_ *β*_1_ = *ζ*_2_ *β*_2_ = *ζ*_3_ *β*_3_ = *ζ*_4_ *β*_4_ = *ζ*_5_ *Ƴ*_*ij*_ = *β*_0*i*_ + *β*_1_ * Time_*j*_ + *β*_2_ * Time_*j*_^2^ + *β*_3_ * Time_*j*_^3^ + *β*_4_ * Time_*j*_^4^ + *ε*_*i*_ *β*_0*i*_ = *ζ*_0_ + *ζ*_0*i*_ * Speed *β*_0_ = *ζ*_1_ * Conversation * Speed * SegmentType *β*_1_ = *ζ*_2_ *β*_2_ = *ζ*_3_ *β*_3_ = *ζ*_4_ *β*_4_ = *ζ*_5_
3. Linear	*Ƴ*_*ij*_ = *β*_0*i*_ + *β*_1_ * Time_*j*_ + *β*_2_ * Time_*j*_^2^ + *β*_3_ * Time_*j*_^3^ + *β*_4_ * Time_*j*_^4^ + *ε*_*i*_ *β*_0*i*_ = *ζ*_0_ + *ζ*_0*i*_ * Speed *β*_0_ = *ζ*_1_ * Conversation * Speed * SegmentType *β*_1_ = *ζ*_2_ * Conversation * Speed * SegmentType *β*_2_ = *ζ*_3_ *β*_3_ = *ζ*_4_ *β*_4_ = *ζ*_5_
4. Quadratic	*Ƴ*_*ij*_ = *β*_0*i*_ + *β*_1_ * Time_*j*_ + *β*_2_ * Time_*j*_^2^ + *β*_3_ * Time_*j*_^3^ + *β*_4_ * Time_*j*_^4^ + *ε*_*i*_ *β*_0*i*_ = *ζ*_0_ + *ζ*_0*i*_ * Speed *β*_0_ = *ζ*_1_ * Conversation * Speed * SegmentType *β*_1_ = *ζ*_2_ * Conversation * Speed * SegmentType *β*_2_ = *ζ*_3_ * Conversation * Speed * SegmentType *β*_3_ = *ζ*_4_ *β*_4_ = *ζ*_5_
5. Cubic	*Ƴ*_*ij*_ = *β*_0*i*_ + *β*_1_ * Time_*j*_ + *β*_2_ * Time_*j*_^2^ + *β*_3_ * Time_*j*_^3^ + *β*_4_ * Time_*j*_^4^ + *ε*_*i*_ *β*_0*i*_ = *ζ*_0_ + *ζ*_0*i*_ * Speed *β*_0_ = *ζ*_1_ * Conversation * Speed * SegmentType *β*_1_ = *ζ*_2_ * Conversation * Speed * SegmentType *β*_2_ = *ζ*_3_ * Conversation * Speed * SegmentType *β*_3_ = *ζ*_4_ * Conversation * Speed * SegmentType *β*_4_ = *ζ*_5_
6. Quartic	*Ƴ*_*ij*_ = *β*_0*i*_ + *β*_1_ * Time_*j*_ + *β*_2_ * Time_*j*_^2^ + *β*_3_ * Time_*j*_^3^ + *β*_4_ * Time_*j*_^4^ + *ε*_*i*_ *β*_0*i*_ = *ζ*_0_ + *ζ*_0*i*_ * Speed *β*_0_ = *ζ*_1_ * Conversation * Speed * SegmentType *β*_1_ = *ζ*_2_ * Conversation * Speed * SegmentType *β*_2_ = *ζ*_3_ * Conversation * Speed * SegmentType *β*_3_ = *ζ*_4_ * Conversation * Speed * SegmentType *β*_4_ = *ζ*_5_ * Conversation * Speed * SegmentType

**Table 2:** Maximum likelihood model comparison in E1

Model	*df*	AIC	BIC	Loglik	Deviance	*Χ*^2^	*df*	*p*
Base	7	− 126,840	− 126,764	63,427	− 126,854			
Intercept	16	− 188,506	− 188,333	94,269	− 188,538	61,684.49	9	< .001***
Slope	23	− 188,658	− 188,408	94,352	− 188,704	165.38	7	< .001***
Quadratic	30	− 188,811	− 188,486	94,435	− 188,871	167.09	7	< .001***
Cubic	37	− 188,810	− 188,409	94,442	− 188,884	12.94	7	0.07365
Quartic	44	− 188,805	− 188,328	94,447	− 188,893	9.46	7	0.22129

*** indicates *p* < .001

**Fig. 3:**
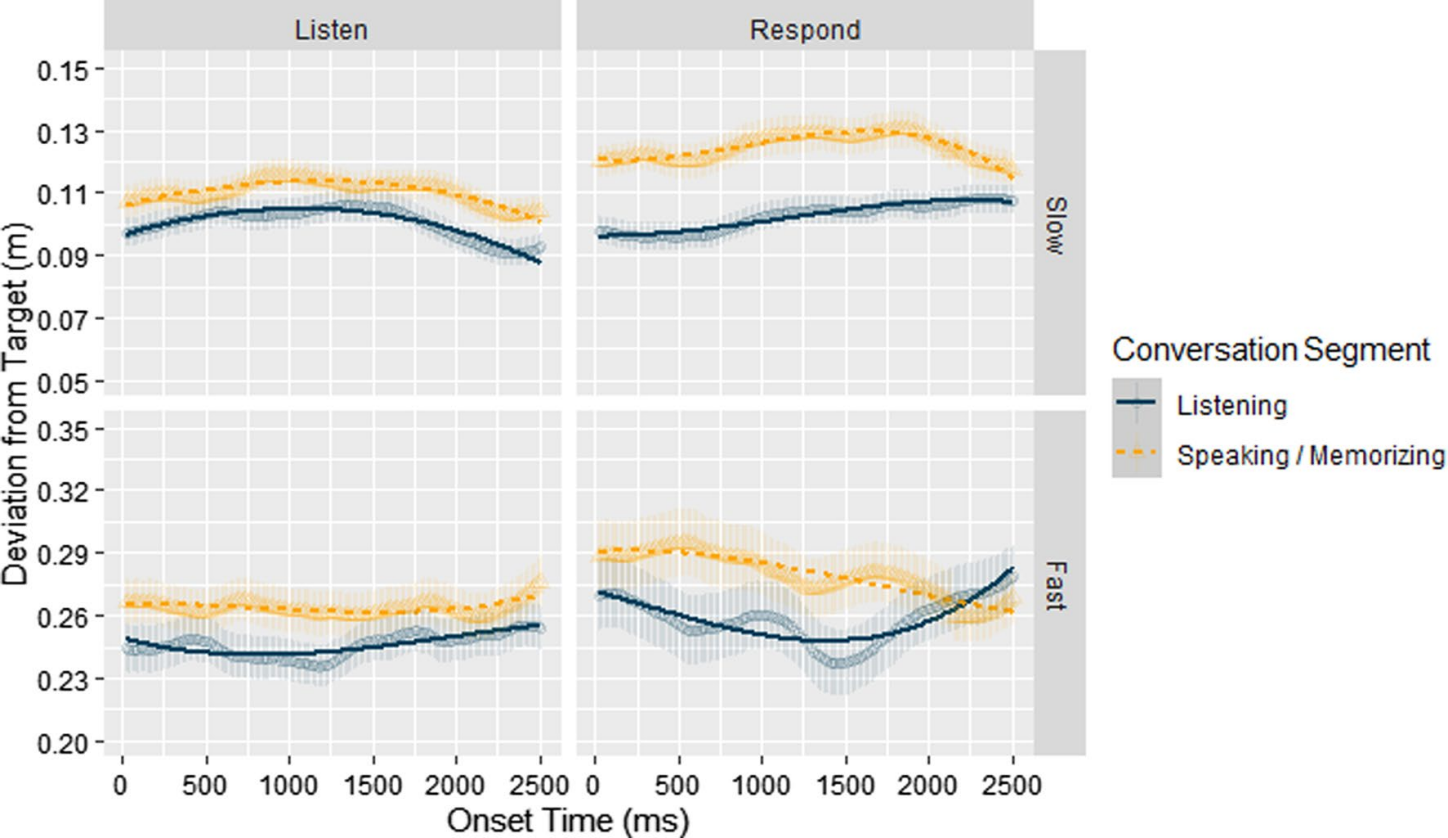
Deviation from target during first 2500 ms of segment onset for Slow and Fast speeds during Listen and Respond conversation conditions in E1. *Error bars* show the SEM. Note that for reasons of graphical clarity, the scales used for the fast and slow target movement conditions are different

As shown in the table, the simplest model that provided a marginally significant better fit of the data than simpler models was the cubic model, *χ*^2^(7) = 12.9371*, p* = 0.07365. The predicted values based on the model are shown in Fig. 3 overlain on the actual data and the coefficients of the model are reported in Table 3 (in Appendix). Inspection of model coefficients and visual inspection of the graph show that coefficients corresponding to all time-independent main effects were significant indicating that: performance was better overall: (1) during Listen conversation blocks compared to Respond conversation blocks; (2) during Slow speed compared to Fast speed conditions; (3) during Listening segments compared to Speaking/Memorizing segments. Likewise, all time-independent interaction effects were also significant showing that (1) performance during listening conditions was slightly worse when participants were memorizing what they heard compared to when they were listening, with a larger effect during fast than during slow speeds, and (2) performance during Respond blocks showed more pronounced differences between Listening and Speaking segments. Most important, as shown by the significant coefficients of the interaction terms that included Time (most notably the 4-way interaction including the quadratic time term) there was a noticeable decrease in performance during the onset of Speaking segments and a gradual increase in performance toward the end of these segments with an opposite effect shown during Listening segments. In this analysis, there were differences between the Listen and Respond conditions, reinforcing our interpretation of the lack of such difference in the former analysis as reflecting the low sensitivity of contrasting the average performance across entire blocks.

**Table 3:** Cubic model coefficients in E1

Fixed effects	Estimate	SE	*df*	*t* value	*p*	
(Intercept)	.1004	.0039	32.36	25.82	< .001	***
Time^1^	− .0286	.0099	379,500	− 2.881	.004	**
Time^2^	− .0430	.0099	379,500	− 4.330	< .001	***
Time^3^	− .0016	.0099	379,500	− 0.157	.875	
Time^4^	− .0004	.0035	379,500	− 0.106	.9158	
conversationrespond	.0019	.0012	379,500	1.593	.1112	
speedfast	.1454	.0071	31.38	20.45	< .001	***
segtypetalk	.0099	.0012	379,500	8.11	< .001	***
conversationrespond:speedfast	.0103	.0017	379,500	5.936	< .001	***
conversationrespond:segtypetalk	.0123	.0017	379,500	7.107	< .001	***
speedfast:segtypetalk	.0080	.0017	379,500	4.623	< .001	***
Time^1^:conversationrespond	.0756	.0141	379,500	5.380	< .001	***
Time^1^:speedfast	.0652	.0141	379,500	4.637	< .001	***
Time^1^:segtypetalk	.0148	.0141	379,500	1.053	.2923	
Time^2^:conversationrespond	.0387	.0141	379,500	2.755	.0059	**
Time^2^:speedfast	.0755	.0141	379,500	5.371	< .001	***
Time^2^:segtypetalk	.0077	.0141	379,500	.545	.586	
Time^3^:conversationrespond	− .0058	.0141	379,500	− .414	.6791	
Time^3^:speedfast	− .0085	.0141	379,500	− .605	.5453	
Time^3^:segtypetalk	− .0015	.0141	379,500	− .104	.9174	
conversationrespond:speedfast:segtypetalk	− .0086	.0025	379,500	− 3.523	< .001	***
Time^1^:conversationrespond:speedfast	− .1023	.0200	379,500	− 5.148	< .001	***
Time^1^:conversationrespond:segtypetalk	− .0504	.0200	379,500	− 2.526	.0115	*
Time^1^:speedfast:segtypetalk	− .0498	.0200	379,500	− 2.507	.0122	*
Time^2^:conversationrespond:speedfast	.0308	.0200	379,500	1.547	.1218	
Time^2^:conversationrespond:segtypetalk	− .0395	.0200	379,500	− 1.980	.0477	*
Time^2^:speedfast:segtypetalk	− .0201	.0200	379,500	− 1.012	.3116	
Time^3^:conversationrespond:speedfast	.0383	.0200	379,500	1.927	.0540	
Time^3^:conversationrespond:segtypetalk	− .0120	.0200	379,500	− 0.603	.5464	
Time^3^:speedfast:segtypetalk	.0223	.0200	379,500	1.121	.2621	
Time^1^:conversationrespond:speedfast:segtypetalk	− .0329	.0282	379,500	− 1.169	.2425	
Time^2^:conversationrespond:speedfast:segtypetalk	− .0685	.0282	379,500	− 2.434	.0149	*
Time^3^:conversationrespond:speedfast:segtypetalk	− .0231	.0282	379,500	− .821	.4117	

* indicates *p* < .05, ** indicates *p* < .01, *** indicates *p* < .001

#### Difficulty rating analysis

**Fig. 4:**
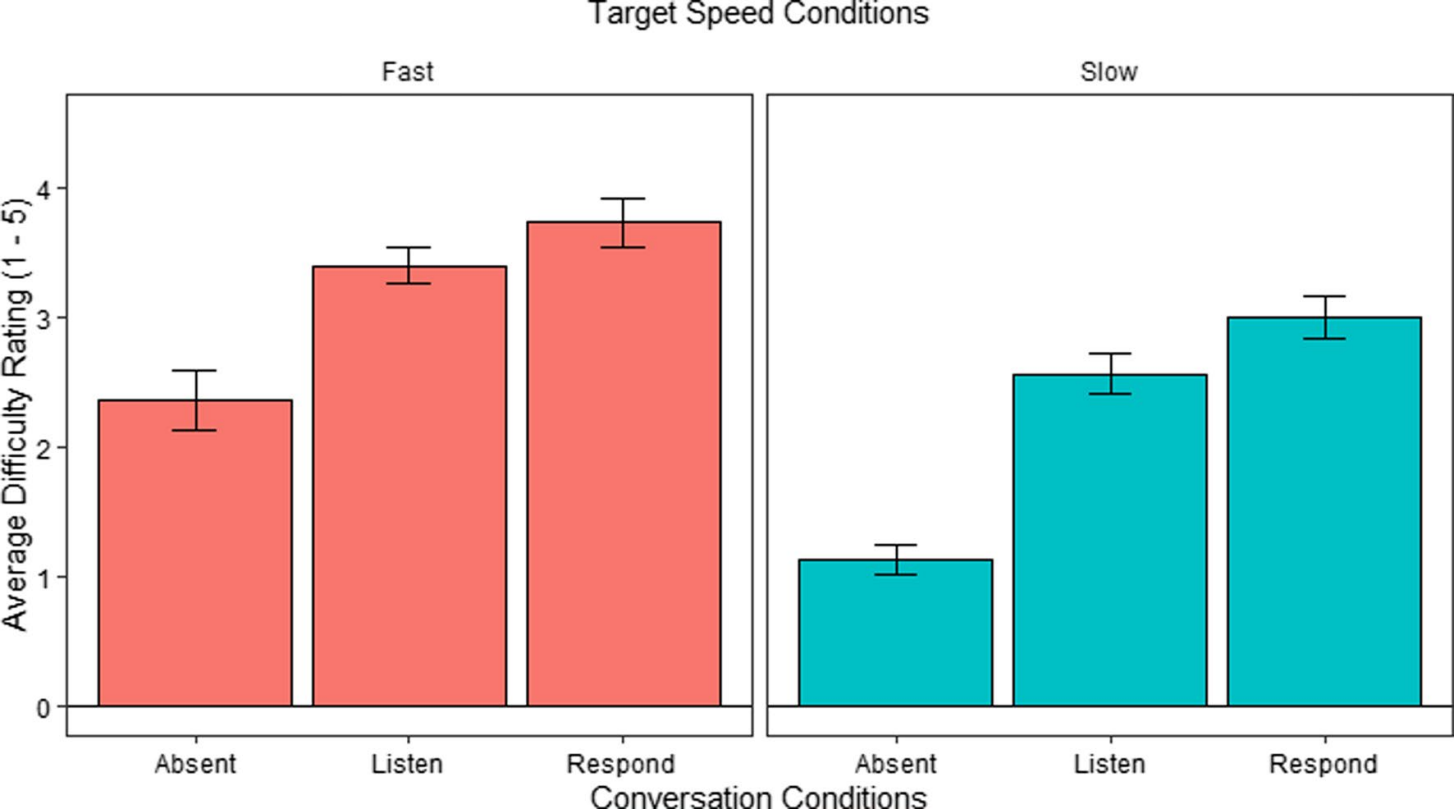
Average difficulty rating per Conversation and Speed conditions in E1 (1—easiest, 5—most difficult). *Error bars* show the SEM

Figure 4 shows the perceived difficulty in the different conditions. We analyzed these using a repeated measures ANOVA to determine whether the difficulty ratings varied as a function of speed and conversation. We found a main effect of Speed, *F*(1, 29) = 54.65*, p* < 0.001, with greater perceived difficulty in the Fast speed conditions compared to the Slow speed conditions. We also found a main effect of Conversation, *F*(2, 58) = 37.80, *p* < 0.001, but no interaction effect, *F* < 1. Follow up post hoc comparisons using Bonferroni correction to explore the main effect of Conversation indicated significant differences between the Absent (*M* = 1.75, *SE* = 0.15) and Listen (*M* = 2.98, *SE* = 0.15) conditions, *t*(58) = − 6.35, *p* < 0.001, and between the Absent and Respond (*M* = 3.37, *SE* = 0.15) conditions, *t*(58) = − 8.32, *p* < 0.001. There were no significant differences between the Listen and Respond conditions, *t* < 2.

#### Recall analysis

**Fig. 5:**
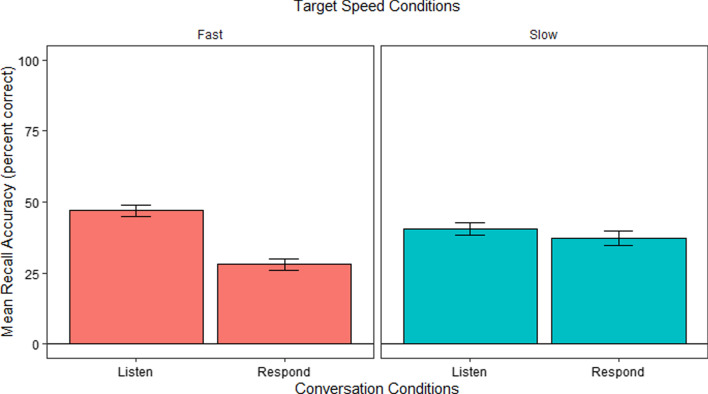
Mean recall accuracy per Conversation and Speed conditions in E1. *Error bars* show the SEM

Figure 5 shows the recall accuracy in the different conditions. We analyzed these using a repeated measures ANOVA to determine whether recall accuracy, measured as the average number of correct survey responses, differed as a function of Speed and Conversation conditions. We found a significant effect of Conversation, *F*(1, 29) = 20.30, *p* < 0.001, such that recall was overall better in the Listen condition than in the Respond condition. We also found a significant interaction between Speed and Conversation, *F*(1, 29) = 9.37, *p* < 0.005. There was no main effect for Speed, *F* < 1. Follow-up post hoc comparisons using Bonferroni correction indicated that the interaction was driven by better recall performance in the Listen (*M* = 0.47, *SE* = 0.03) than in the Respond (*M* = 0.28, *SE* = 0.03) conditions only during the Fast conditions, *t*(57.9) = 5.33*, p* < 0.001 but not during the Slow conditions, *t* < 1.

### Discussion

Our first critical hypothesis, H1, stated that performance should change dynamically throughout the course of conversation with performance being best at the beginning of listening segments, then gradually decreasing during speaking and memorizing conversation segments, and that, importantly, these effects will be more pronounced in the responding blocks than in the listening blocks. In support of this hypothesis, the GCA time-course analyses revealed the predicted gradual decline in performance during listening segments and improved performance during speaking and memorizing segments, and this decline was strongest in the Fast target speed and Respond conditions.

Our second critical hypothesis, H2, stated that variation in tracking and recall performance due to conversation complexity in the different target speed conditions should reveal whether the load associated with increased tracking speed is perceptual or cognitive. According to Lavie et al.’s (2004) load theory, more attentional resources are available to *process* distracting stimuli when perceptual load is low, while fewer resources are available when perceptual load is high or at capacity. At the same time, the theory suggests that more attentional resources are available to *reject* distracting stimuli when cognitive load is low, while this ability diminishes as cognitive load increases. In our case, we hypothesized that differences in the effect of conversation complexity on tracking performance between slow and fast target speeds should reveal whether the interference between driving and conversation reflects perceptual or cognitive loads. If perceptual load drives the interference, conversational complexity should have a stronger effect in the slower conditions than in the faster conditions where fewer resources would be available to process the conversation. Alternatively, if fast speeds increase cognitive and not perceptual load, in comparison with slow speeds, changes in tracking performance due to conversation complexity should be less noticeable in the slow compared to the fast speeds because more cognitive resources are available for processing the distracting conversation in the slow speeds. The results from the overall analysis showed that during slow speeds, performance did not significantly change across conversation conditions, while in fast speeds it worsened as conversation became more difficult. These results were reinforced by the more sensitive GCA analyses, which found differences between the conversation conditions for all speeds but revealed that these differences were greater for the faster speeds. Consistent with the tracking data, recall results showed no difference between the listening and responding conditions during slow speeds and better recall in the Listen than Respond condition during fast speeds, indicating poorer retention of verbal information in the Fast speed and Respond condition. While it is possible that the absence of differences in the different measures in the Slow speed conditions reflects low power, our emphasis here is on the interactions and specifically that these differences were clearly stronger in the fast conditions. Therefore, regardless of whether effects in the Slow conditions may be revealed by a more powerful design, and in line with H2, our results show that the interference between driving and conversation likely reflects increased demands for cognitive rather than perceptual resources.

With respect to our more general predictions, as expected, tracking a fast-moving target was more demanding than tracking a slow-moving target. Further supporting this finding, GCA time-course analyses showed that performance was worse throughout conversation conditions for all conversation segments during fast speeds compared to slow speeds, and for the speaking and memorizing conversation segments compared to listening segments. As for our other general prediction, the analysis of difficulty ratings showed that performance in the Absent conversation condition was rated as less difficult than both the Listen and Respond conditions, while perceived difficulty was similar for both the Listen and Respond conditions. Likewise, and as expected, difficulty ratings were higher overall for fast speeds compared to slow. These findings, while not very surprising, are nevertheless important in demonstrating that target moving speed and the presence of conversation modulate perceived task difficulty, affirming the effectiveness of our manipulations.

While we did not make any predictions about the recall results, it is interesting to note that we did observe differences between conditions such that recall was overall better following listening blocks than the responding blocks, with this difference showing significantly in the Fast but not Slow conditions. As there could be several possible explanations for this finding that our data cannot distinguish, we will leave for future research the exploration of the effects of the dual task on memory retention.

In summary, the results from E1 show that the tracking task performance deteriorated with increased difficulty, which was modulated by changes in speed as well as by the presence or absence of verbal conversation tasks. While the differences between speaking and listening were less robust than predicted in both the overall analysis of driving performance and in the analysis of perceived difficulty, these differences were detected in the more sensitive analysis of the conversational segments. This may indicate that the finer demands of verbal conversation may only be detected during difficult conditions or more sensitive analyses. In the next experiment, we examine a situation that makes our task more difficult by involving the visual modality as part of the conversation task. We expect that the overall greater difficulty will enhance the effects we found in this experiment.

## Experiment 2

According to Wickens (2002), interference between tasks reflects the overlap between their demands in different modalities. In E2, we presented verbal stimuli using the visual modality expecting that the higher overlap between the modalities of the verbal and tracking tasks would result in even stronger interference. Specifically, E2 tested tracking performance during fast and slow target speeds, and under conditions involving no verbal tasks (Absent), conditions with reading written prompts overlain on the driving simulator screen (Read), and conditions in which participants responded to the written prompts (Respond). We believe this is akin to reading text messages while performing certain aspects of driving since both sets of tasks can heavily involve continuous visual-spatial processing.

Our hypotheses for E2 were similar to those we had for E1. H1 was that performance would change dynamically throughout the course of conversation with performance being best at the beginning of reading segments and then gradually decrease during planning and speaking segments. H2 was that variation in tracking performance would reveal whether the load associated with increased tracking speed is perceptual or cognitive. In addition, we also hypothesized that, due to the use of overlapping visual modality for the tracking and reading tasks, the reading manipulation in E2 would result in more pronounced interference (H3).

Our general predictions for E2 also closely mirror those for E1: driving performance would be more prone to interference from conversation during fast speeds than during slow speeds; performance would be best when no conversation is present, second best when reading written text, and worst when verbally responding to the read text; perceived difficulty would be worse in fast compared to slow speeds; and that perceived difficulty would be lowest in the absent conditions, higher in the reading conditions, and highest in the responding conditions.

We again included a recall task to encourage participants to process the verbal stimuli, but as our focus here is on the effect conversation has on driving, we make no prediction about post-block recall performance.

### Methods

#### Participants

A total of 31 participants (age: *M* = 20.10, *SD* = 1.51) from the University of South Carolina Department of Psychology undergraduate participant pool took part in the study. Of the 31 participants, there were 6 males (age: *M* = 20.50, *SD* = 1.63) and 25 females (age: *M* = 20.02, *SD* = 1.49).

#### Procedure

The procedure was the same as E1, except that instead of Listen conditions E2 had Read conditions.

#### Data preparation

The data preparation was the same as E1, except that instead of Listen conditions and Listening segments, E2 had Read conditions and Reading segments.

#### Conversation task

**Fig. 6:**
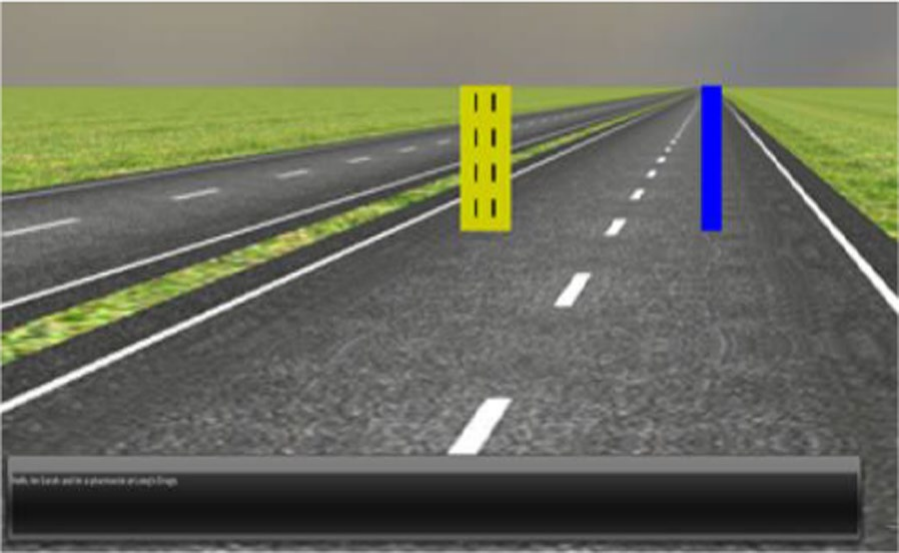
While participants perform the primary tracking task, text prompts are presented at the bottom of the driving simulator screen

During a Conversation condition (both Read and Respond conditions), participants were exposed to 12 written statements at a rate of about one per every 15 s via text overlain on the simulator screen (Fig. 6). The texts were presented in Arial 12 font at the bottom-left corner of the screen for approximately six seconds, and their precise onsets were jittered to prevent participants from predicting when each will be shown. This text size was clearly visible and easily readable for participants.

### Results

Examination of the video recordings of experimental sessions revealed that one participant did not correctly comply with the instructions and thus their results were excluded from the analysis. The data from the remaining 30 participants (age: *M* = 20.13, *SD* = 1.53) were submitted for further analyses. Of these, six were male (*age: M* = 20.50*, SD* = 1.63) and 24 female (age: *M* = 20.04*, SD* = 1.49).

#### Overall analysis

**Fig. 7:**
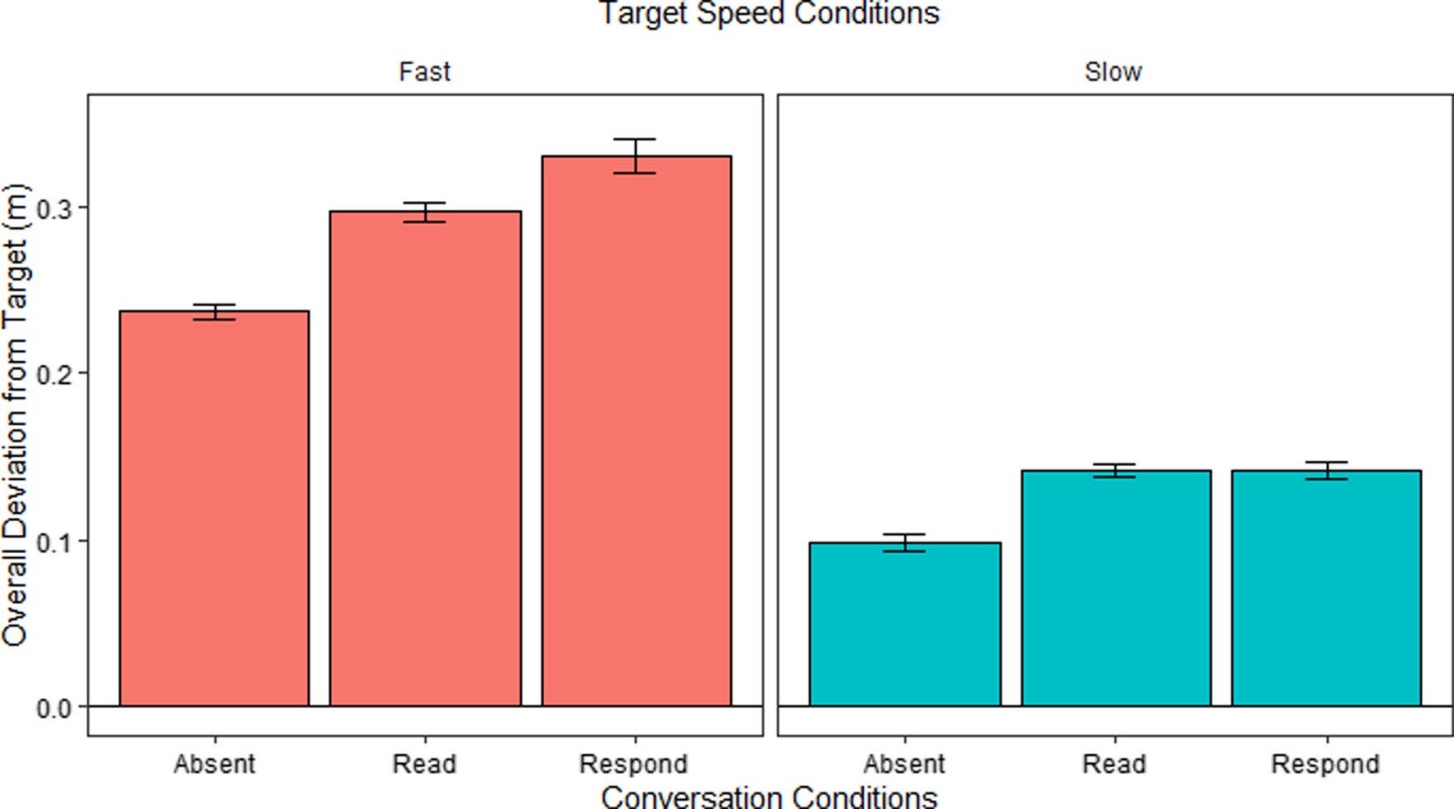
Overall deviation from target during different conversation conditions in Fast and Slow speed conditions in E2. *Error bars* show the SEM. Note that for graphical clarity, the scales of this figure are different than those used in Fig. 2

Figure 7 shows the overall absolute deviation in meters from target (Deviation) in the different conditions. We analyzed these using a repeated measures ANOVA with Speed and Conversation set as within-subject factors, and found significant main effects for both Speed, *F*(1, 29) = 603.43*, p* < 0.001, and Conversation, *F*(2, 58) = 72.75, *p* < 0.001, as well as an interaction between Speed and Conversation, *F*(2, 58) = 11.38*, p* < 0.001.

To better understand the nature of the 2 × 3 interaction, we followed up with Bonferroni corrected post hoc comparisons of performance in the conversation conditions separately for the Fast and Slow conditions. For the Fast conditions, there were significant differences between the Absent and Read conditions, *t*(115) = − 7.52, *p* < 0.001, Absent and Respond conditions, *t*(115) = 11.69, *p* < 0.001, and Read and Respond conditions, *t*(115) = 4.17, *p* < 0.001. In the Slow conditions, there were significant differences between Absent and Read conditions, *t*(115) = − 5.52, *p* < 0.001, and Absent and Respond conditions, *t*(115) = 5.41, *p* < 0.001. The difference between Read and Respond conditions was not significant, *t* < 2.

#### Time-course analysis

Similar to E1, we used GCA’s to analyze the first 2500 ms (i.e., 133 samples) of each conversation segment. We used similar random-coefficient model equation structure as in E1 (Table 1, in Appendix), with maximum likelihood estimates and Akaike information criterion (Long, 2012) (Table 4), and report the selection criteria for the models we compared and include graphs that show the predicted values based on the chosen model, and the fitted data.

**Table 4:** Maximum likelihood model comparison in E2

Model	*df*	AIC	BIC	Loglik	Deviance	*Χ*^2^	*df*	*p*
Base	7	195,163	195,239	− 97,575	195,149			
Intercept	16	137,966	138,140	− 68,967	137,934	57,214.82	9	< .001***
Slope	23	125,513	125,763	− 62,734	125,467	12,467.19	7	< .001***
Quadratic	30	125,332	125,657	− 62,636	125,272	195.38	7	< .001***
Cubic	37	125,075	125,476	− 62,500	125,001	270.85	7	< .001***
Quartic	44	125,047	125,524	− 62,479	124,959	42.19	7	< .001***

*** indicates *p* < .001

**Fig. 8:**
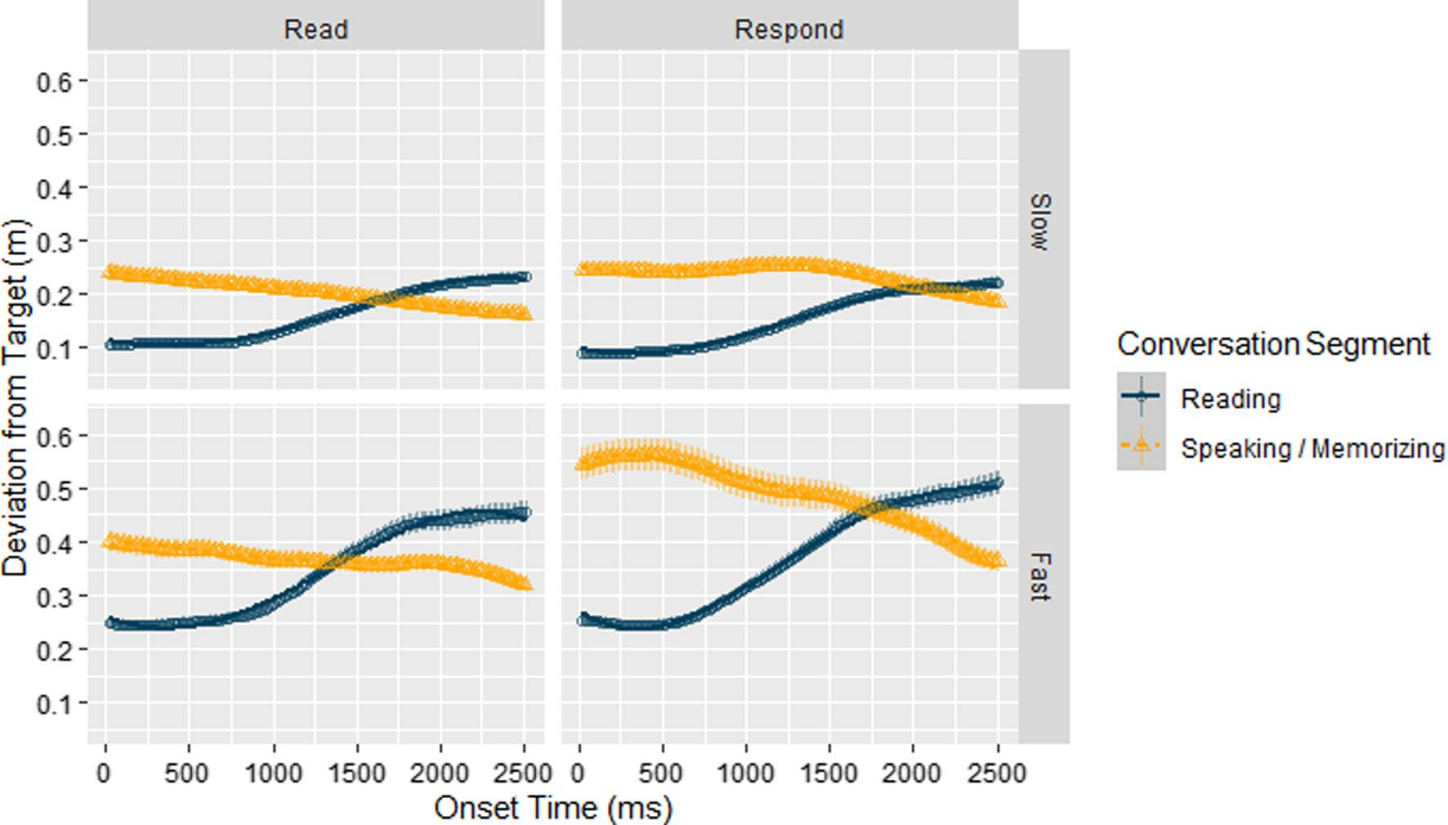
Deviation from target during first 2500 ms of segment onset for Slow and Fast speeds during Read and Respond conversation conditions in E2. *Error bars* show the SEM. Note that for reasons of graphical clarity, the scales of this figure are different than those used in Fig. 3

As shown in the table, the quartic model provided significantly better fit than simpler models, *χ*^2^(7) = 267.13*, p* < 0.001. The predicted values based on the model are shown in Fig. 8 overlain over the actual data. The coefficients of the model are reported in Table 5 (in Appendix). Inspection of model coefficients and visual inspection of the graph show a pattern of results that is much clearer and more aligned with our hypothesis than in E1. The coefficients corresponding to all time-independent main and interaction effects were highly significant. This shows that performance was better overall: (1) during Read conversation blocks compared to Respond conversation blocks; (2) during Slow speed compared to Fast speed conditions; (3) during Reading segments compared to Speaking/Memorizing segments; (4) performance during Read conditions was slightly worse when participants were memorizing what they heard compared to when they were reading, with a larger effect during fast than during slow speeds; and (5) performance during Respond conditions showed more pronounced differences between Reading and Speaking segments. Most important, as shown by the significant coefficients of the interaction terms that included Time (most notably the 4-way interactions including the linear and quartic time terms, respectively) there was a noticeable decrease in performance during the onset of Speaking segments and a gradual increase in performance toward the end of these segments with an opposite effect shown during Reading segments, but this change was rather abrupt and clearly not linear. Again, the results of this experiment much more clearly align with our hypotheses further reinforcing our assumption that the predicted effects will be easier to detect under more demanding tasks.

**Table 5:** Quartic model coefficients in E2

Fixed effects	Estimate	SE	*df*	*t* value	*p*	
(Intercept)	.1599	.0097	30.84	16.55	< .001	***
Time^1^	.5322	.015	379,400	35.41	< .001	***
Time^2^	.0689	.015	379,400	4.584	< .001	***
Time^3^	− .1052	.015	379,400	− 6.998	< .001	***
Time^4^	− .0171	.015	379,400	− 1.14	.2544	
conversationrespond	− .0083	− .0018	379,400	− 4.48	< .001	***
speedfast	.1829	.0125	310,200	14.65	< .001	***
segtypetalk	.0416	.0019	379,400	22.49	< .001	***
conversationrespond:speedfast	.0334	.0026	379,400	12.77	< .001	***
conversationrespond:segtypetalk	.041	.0026	379,400	15.66	< .001	***
speedfast:segtypetalk	− 0184	.0026	379,400	− 7.05	< .001	***
Time^1^:conversationrespond	.0266	.0213	379,400	1.251	.2108	
Time^1^:speedfast	.3986	.0213	379,400	18.75	< .001	***
Time^1^:segtypetalk	− .7984	.0213	379,400	− 37.56	< .001	***
Time^2^:conversationrespond	− .0515	.0213	379,400	− 2.423	.0154	*
Time^2^:speedfast	− .0214	.0213	379,400	− 1.007	.3142	
Time^2^:segtypetalk	− .083	.0213	379,400	− 3.905	< .001	***
Time^3^:conversationrespond	− .0091	.0213	379,400	− .43	.6672	
Time^3^:speedfast	− .1150	.0213	379,400	− 5.413	< .001	***
Time^3^:segtypetalk	.1095	.0213	379,400	5.151	< .001	***
Time^4^:conversationrespond	.0173	.0213	379,400	.812	.4170	
Time^4^:speedfast	.0046	.0213	379,400	.218	.8276	
Time^4^:segtypetalk	.028	.0213	379,400	1.317	.1879	
conversationrespond:speedfast:segtypetalk	.0553	.0037	379,400	14.94	< .001	***
Time^1^:conversationrespond:speedfast	.1554	.0301	379,400	5.171	< .001	***
Time^1^:conversationrespond:segtypetalk	.0704	.0302	379,400	2.332	.0197	*
Time^1^:speedfast:segtypetalk	− .3271	.0301	379,400	− 10.88	< .001	***
Time^2^:conversationrespond:speedfast	.0338	.0301	379,400	1.124	.2608	
Time^2^:conversationrespond:segtypetalk	− .0721	.0302	379,400	− 2.291	.0168	*
Time^2^:speedfast:segtypetalk	.0037	.0301	379,400	1.253	.2103	
Time^3^:conversationrespond:speedfast	− .0037	.0301	379,400	− .123	.9022	
Time^3^:conversationrespond:segtypetalk	− .0277	.0301	379,400	− .92	.3577	
Time^3^:speedfast:segtypetalk	.0736	.0301	379,400	2.447	.0144	*
Time^4^:conversationrespond:speedfast	.0343	.0301	379,400	1.14	.2544	
Time^4^:conversationrespond:segtypetalk	.0181	.0302	379,400	.601	.5480	
Time^4^:speedfast:segtypetalk	− .0570	.0301	379,400	− 1.897	.0578	
Time^1^:conversationrespond:speedfast:segtypetalk	− .7318	.0426	379,400	− 17.18	< .001	***
Time^2^:conversationrespond:speedfast:segtypetalk	− .0491	.0426	379,400	− 1.152	.2493	
Time^3^:conversationrespond:speedfast:segtypetalk	.0783	.0426	379,400	1.838	.066	
Time^4^:conversationrespond:speedfast:segtypetalk	− .0898	.0426	379,400	− 2.109	.0349	*

* indicates *p* < .05, *** indicates *p* < .001

#### Difficulty rating analysis

**Fig. 9:**
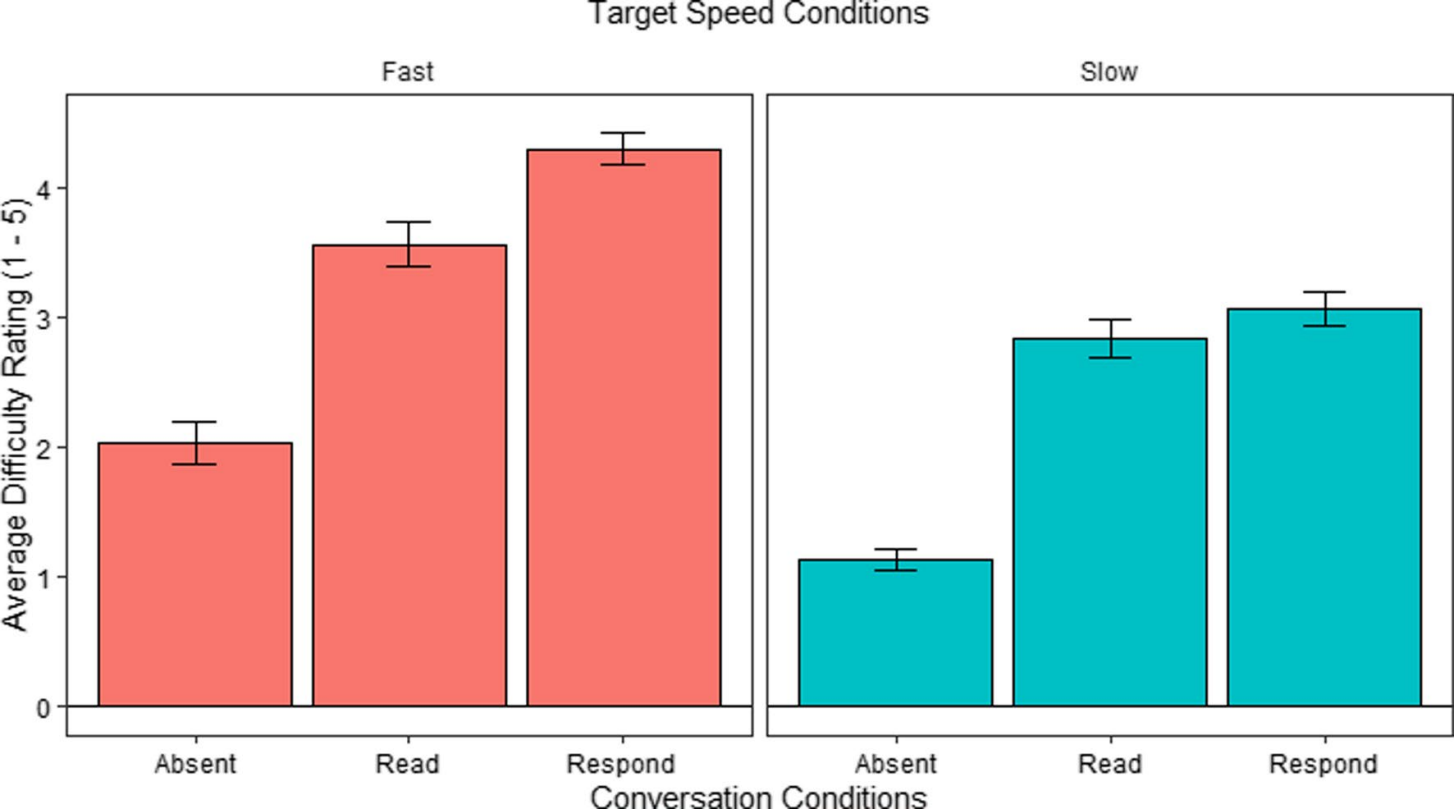
Average difficulty rating per conversation and speed conditions in E2 (1—easiest, 5—most difficult). *Error bars* show the SEM

Figure 9 shows the perceived difficulty ratings in the different conditions. We analyzed these using a repeated measures ANOVA to determine whether the difficulty ratings of each block differed as a function of Speed and Conversation. We found main effects of both Speed, *F*(1, 29) = 44.83, *p* < 0.001, and Conversation, *F*(2, 58) = 130.77, *p* < 0.001, but no interaction effect, *F* = 2.06. Follow up post hoc comparisons using Bonferroni correction to explore the main effect of conversation indicated significant differences between the Absent (*M* = 1.58, *SE* = 0.11) and Read (*M* = 3.20, *SE* = 0.11) conditions, *t*(58) = − 11.89, *p* < 0.001 the Absent and Respond (*M* = 3.68, *SE* = 0.11) conditions, *t*(58) = − 15.44, *p* < 0.001, and the Read and Respond conditions, *t*(58) = − 3.55,* p* = 0.002. Again, the current experiment showed stronger results than the previous one in that here even the difference between the Read and Respond conditions was significant. As with the previous analyses, this finding reinforces our assumption that detecting the hypothesized results requires the combination of the two tasks to be difficult enough.

#### Recall analysis

**Fig. 10:**
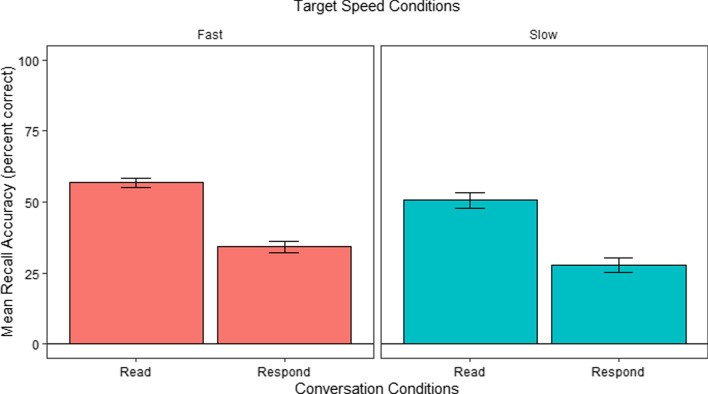
Mean recall accuracy per Conversation and Speed conditions in E2. *Error bars* show the SEM

Figure 10 shows the average recall accuracy in the different conditions. We analyzed these using a repeated measures ANOVA to determine whether recall accuracy differed as a function of Speed and Conversation conditions. We found significant effects for Speed, *F*(1, 29) = 8.32, *p* < 0.007, and Conversation, *F*(1, 29) = 97.28, *p* < 0.001. There was no interaction effect, *F* < 1. This result is different than in E1 where recall performance in the Respond conditions was no different than the Listen conditions in the Slow speeds and better in the Fast conditions. We do not have a ready explanation for the better recall in the Fast conditions than the Slow ones in this experiment or for the different patterns of recall performance in the two experiments and thus leave it for future research to disentangle.

### Discussion

As we expected, the results of E2 resembled those of E1 but showed the predicted effects more clearly. H1 received strong support in this experiment in which the predicted dynamic changes in performance associated with the different conversational conditions were strongly attested. Very clearly, and just as we predicted, these changes were stronger in the more difficult Fast conditions than in the easier Slow conditions. Furthermore, GCA time-course analyses revealed that performance was best at the beginning of the reading segments, then abruptly decreased during the speech planning and production segments, while performance was worst at the beginning of speaking segments then abruptly improved during the course of speech. Additionally, greatest variation was found in the responding and Fast speed conditions. These effects were similar to results found during the Fast and Respond conditions in E1, except they were found for all conditions in this experiment. This fully supports H1.

Regarding H2, the results of this experiment likewise reinforce those of E1. There was a greater effect of conversational complexity in the higher speeds than in lower speeds, supporting the conclusion that the interference between tracking performance and the verbal task was driven by increased cognitive rather than perceptual load (Lavie, et al., 2004).

Our third hypothesis for this experiment (H3) was that the overlapping visual requirements of the tracking and reading tasks, E2 would result in more pronounced interference than E1. This was clearly the case as is apparent by looking at the data patterns in both experiments, which are similar yet more pronounced in E2.

With respect to our other predictions, as in E1, tracking performance was worse in the fast speeds compared to slow speeds in each conversation condition. Additionally, during fast speeds, performance was best when verbal tasks were Absent, worse during Read conditions, and worst during the Respond conditions. Performance during slow speeds, however, was significantly worse in the two conversation present conditions (Read and Respond) compared to when conversation was absent, with no significant difference in performance during Read and Respond conditions. Also, similar to E1, the analysis of difficulty rating showed that performance in the Absent verbal task condition was rated as least difficult, the Read condition as more difficult, and the Respond condition as most difficult for both fast and slow target speeds. As expected, ratings were higher overall for fast speeds compared to slow.

In summary, the results from E2 show that the difficulty of the tracking task increased as participants simultaneously read prompts overlain on the driving simulator screen. Similar to E1, this difficulty was modulated by changes in speed as well as by whether participants had to respond or not. The differences between speaking and reading in this experiment were clearly more robust than the difference between speaking and listening in E1, especially for fast speeds. This may indicate that the finer demands of verbal conversation can only be detected during difficult conditions and may support that the lack of effects in the Slow conditions of E1 may reflect low power. Overall, these results fully support H3, and show that the visual demands of reading text highly interfere with those of tracking. This likely reflects the intra-modal time-sharing between the reading and tracking tasks in this experiment, compared to the cross-modal time-sharing between tracking and listening tasks in E1 (Liu & Wickens, 1989; Recarte & Nunes, 2003; Wickens, 2008). Note that it may also be the case that the interference in this experiment merely reflects the requirement to divert eye gaze and attention from the driving part of the screen to the text window. While this can perhaps explain the overall worse performance in the Fast conditions than the Slow conditions, it cannot explain the interaction between the Speed and Conversation conditions. This is because if visual distraction were the only factor underlying performance in this experiment, it should have affected the Read and Respond conditions equally.

## General discussion

In this paper, we reported two experiments that utilized a novel driving simulator paradigm that we developed to capture the fine-grain changes in the demands of multitasking involving language processing on tracking performance during driving. Both experiments measured performance under conditions in which the difficulty of the tracking task and the requirements of the verbal task were manipulated. The modality of the verbal task varied across experiments, with the first experiment focusing on the auditory modality and the second experiment utilizing the visual modality for presenting the verbal prompts.

The primary task in this study was the ConTRe (Mahr et al., 2012) smooth pursuit tracking implemented within the OpenDS (Math et al., 2012) driving simulator environment. As we hoped, this paradigm allowed us to measure the effects of a concurrent interactive verbal task at a high temporal resolution and thus provided a critical test of a psycholinguistic explanation of the well-known interference between conversation and tracking performance during driving.

In order to manipulate the difficulty of the main tracking while driving task, we controlled the speed of the moving target so that participants performed the tracking task under Fast and Slow speed conditions. In both experiments, we found that tracking performance was worse during blocks in which target speed was fast compared to when it was slow. Therefore, our speed manipulation was effective in modulating overall task difficulty. We attribute this to the increased demands that fast target speeds place on visual-motor resources while driving.

Then, to capture the effects of conversation on the primary tracking task, we manipulated conversation type so that participants either tracked without engaging in any verbal task (both E1 and E2) or listened to prerecorded verbal statements and responded to what they heard (E1), or read written prompts and responded to what they read (E2). In both experiments, we found that performance on the tracking task was worse overall in conditions in which a verbal task was present compared to when there was no verbal task. This result replicates previous findings about interference between conversation and driving (Strayer & Drews, 2007; Strayer et al., 2015).

Next, we looked at how performance changed as a function of target speed and conversation type to determine whether different aspects of verbal tasks pose different demands on tracking while driving. Specifically, we contrasted the effect of different conversation conditions (absent vs. listen/read-only vs. respond) on tracking under the two speed conditions. In E1, increased speeds caused worse performance when conversation was present compared to when it was absent. However, there was not a difference between the two conversational conditions (listen-only vs. respond) when performance was averaged across a several minute long block. In E2, increased speeds also caused worse performance when participants read text compared to when verbal tasks were absent, but performance was even worse when participants were also required to respond to what they read. Overall, these results show that language production is more demanding than language comprehension, but that these effects become detectable only under difficult situations where the demands of both tasks are high and/or employ overlapping modality (Wickens, 2002).

Most importantly, we utilized GCAs to assess the fine grain dynamic changes in performance at the beginning of each conversation segment as participants listened to speech, read verbal prompts, and either memorized or planned and produced speech in response to what they heard or read. These analyses revealed that, in both the listening and responding blocks in E1, and, more strongly, in both the reading and responding blocks in E2, performance gradually degraded during listening and reading segments and gradually improved during responding segments. This reflects dynamic changes in the demands of the verbal tasks that are consistent with psycholinguistic theories of comprehension and production (Hoey & Kendrick, 2017; Lee et al., 2017; Pickering & Garrod, 2013). While listening and reading may not require many resources to begin with, once production planning commences toward the end of these segments, resource demands increase. Conversely, while responding requires many resources to begin with as planning goes on while speaking, once planning wraps up toward the end of these segments, resource demands decrease. The overall greater difficulty of the verbal task in E2 than in E1, made that pattern more pronounced.

Recall tests administered at the end of each block assessed how well participants retained the verbal information they heard or read during the block. In E1, recall performance was better in the listening conditions than in the responding conditions during fast speeds, but not significantly different between the two conditions during slow speeds. In E2, recall was better in the reading compared to the responding conditions in both speeds, and better during fast compared to slow speeds in both conversation conditions. In addition, recall performance was overall better in E1 than in E2, consistent with E2 being more demanding than E1. Recall performance also showed variation between conditions that was different in the two experiments. Since this task was only included as means to ensure participants processed the verbal material and was not the primary focus of this research, and since the patterns of these data may reflect different underlying mechanisms that our data cannot disentangle, we leave a more detailed exploration of the recall findings for future research.

We also collected the participants’ perceived difficulty at the end of each block. In both experiments, participants rated conditions with fast target speeds as more difficult than those with slow target speeds, and conversation blocks as more difficult than blocks with no conversation. However, in E1, perceived difficulty did not differ between the two conversation conditions, while in E2, participants perceived the responding conditions to be more difficult than the reading conditions during fast speeds. Similar to recall performance, perceived difficulty appears to reflect the cumulative difficulty of both tasks. The overall greater difficulty of the reading task in E2 than the listening task in E1 allowed the difficulty difference between the listening/reading conditions and the responding conditions to affect participants’ conscious perceived difficulty. However, speed and conversation conditions did not interact, even in the more difficult E2. The difference between the subjective ratings and actual tracking performance highlights the limits of participants’ awareness of their own tracking performance. We return to this point when discussing the practical implications of our findings in the Conclusion section.

### Model of concurrent driving and conversation

**Fig. 11:**
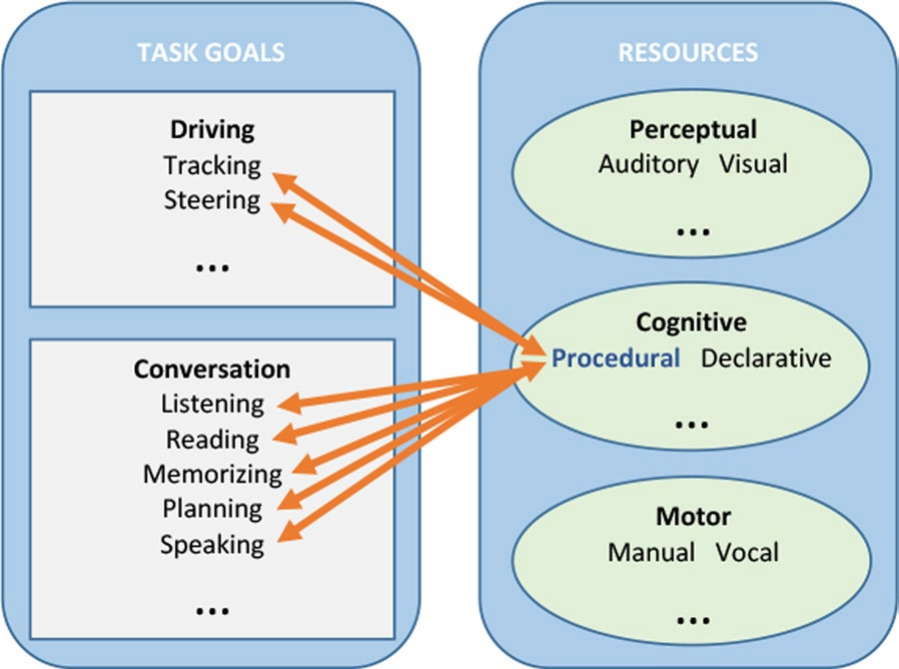
Model of driving and conversation. Task goals and resources are coordinated, managed, and executed by a central procedural resource (following Salvucci & Taatgen, 2008)

Having shown that performing a lower-level routine task that is critical for driving is sensitive to subtle language processing demands, we now describe our results in terms of a general model of multitasking and resource allocation (Fig. 11).

The details of our model closely follow Salvucci and Taatgen (2008) with the addition of driving and conversation tasks as independent task goals that continuously make requests for attentional resources during concurrent task execution. These requests are managed and processed during 50 ms intervals by a central procedural resource that taps resources according to task goal requirements and resource availability. During concurrent driving and conversation, this resource alternates processing between task goals, so that processing on one task goal must be initiated before processing can start on another. Once initiated, however, both task goals and resources can be processed in parallel if conflicts do not occur, such as when one task goal must wait for another task to release a needed resource (i.e., procedural and peripheral bottlenecks). Therefore, our model borrows from Salvucci and Taatgens’ (2008) threaded cognition framework to account for how task goals are coordinated and executed during concurrent driving and conversation and Wickens’ (2002) multiple resource theory to describe the shared attentional resources tapped by these tasks.

Further, our model can provide a useful means of predicting performance at a fine grain time scale not previously modeled in the literature. Specifically, it can account for the dynamic shifting patterns in performance found in our study when participants concurrently engage in multimodal conversation tasks. It does this by representing the interleaving processing of task goals and resources during the course of each conversation task. During less demanding conversation tasks (e.g., listening and speaking in E1) more attentional resources may be available to be shared between interleaved tasks, resulting in fewer processing conflicts and better performance during these intervals. However, during more demanding tasks (e.g., reading and speaking in E2), less attentional resources may be available, resulting in more processing conflicts and worse performance during these intervals. Further, the involvement of the central procedural resource in processing of task goals also supports the role of cognitive demand on task performance discussed in Lavie et al.’s (2004) load theory. Thus, the predictions from our model fit well with the results discussed in this study. Importantly, this model, which is only described here in very general terms, can be easily extended to make predictions about other aspects of conversation that can likely affect driving performance such as the content of the conversation, the linguistics complexity of the input, etc. (Demberg & Sayeed, 2016; Demberg et al., 2013; Funk et al., 2020).

### Limitations

Clearly our study has several important limitations. First, it could be argued that the ConTRe task may not be sufficiently representative of actual driving performance. While this is a valid concern, we believe that it does capture an important aspect of driving, namely routine steering performance while driving. There is indeed considerable research showing the utility of this task for studying driving performance (Demberg, 2013; Häuser et al., 2019; Rajan et al., 2016; Reichel et al., 2014; Vogels et al., 2018). There is also considerable research recognizing the importance of combining studies of natural driving with better controlled lab-based studies in order to establish a complete picture of the factors underlying driving performance (Boyle & Lee, 2010; Bruck et al., 2020; Caird et al., 2014a, 2014b; Guo, 2019; Underwood et al., 2011; Wijayaratna et al., 2019).

Another potential limitation of our study may be lack of sufficient power, especially in Experiment 1. However, since our focus here was on the interaction between the effect of conversation and the effect of tracking speed, which we observed in most measures in both experiments, we do not think that this concern limits the implications of our results. Nevertheless, we acknowledge that repeating Experiment 1 with more participants might reveal conversational differences even in the slower speeds.

Finally, our sample consisted of more females than males. Given well known sex differences (e.g., Kaufman, 2007; Mathew et al., 2020; Murray et al., 2018) in psychophysical tasks such as the tracking task used here, it may be the case that the effects we observed are more representative of females than males. While this is an interesting possibility that should be addressed in future research, we do not think that it reduces the importance of our findings.

## Conclusion

In this article, we showed that different aspects of verbal conversation can negatively affect performance on a driving-based tracking task. The results from our study are consistent with load-based theories of multitasking performance and show that language planning and language production, and, to a lesser extent, language comprehension tap similar resources as those used for lateral vehicle control, an important and relatively low-level aspect of driving that may seem to be independent of the temporal requirements of language processing. Additionally, our work shows that growth curve analyses can provide an effective means of capturing the dynamic changes in performance over time due to rapid changes in resource requirements predicted by psycholinguistic theories to be associated with specific aspects of conversation. The paradigm we developed provides a simple and easy means of testing theoretical models of driving, language comprehension and production, attentional resource allocation, and multitasking. As such, this paradigm and the data we collected provide a solid basis for future studies on the resource requirements of other aspects of language processing and their influence on driving in various contexts. As the difficulty ratings we collected show, participants, and thus drivers, are unaware of their actual performance, suggesting the need for educational and perhaps technological interventions to increase driving safety while engaging in conversation. Specifically, technological efforts might concentrate on identifying in person or remote conversation involving the driver and increase attention and vigilance interventions as drivers get ready to speak or are speaking. This work also reinforces the importance of reducing the overlap between modalities used for presenting information to drivers and those used for driving.

## Data Availability

The datasets used and analyzed during the current study are available from the corresponding author on reasonable request.

## Appendix

See Tables 1, 3 and 5.
